# P1 Bacteriophage-Enabled Delivery of CRISPR-Cas9 Antimicrobial Activity Against *Shigella flexneri*

**DOI:** 10.1021/acssynbio.2c00465

**Published:** 2023-02-20

**Authors:** Yang W. Huan, Vincenzo Torraca, Russell Brown, Jidapha Fa-arun, Sydney L. Miles, Diego A. Oyarzún, Serge Mostowy, Baojun Wang

**Affiliations:** † College of Chemical and Biological Engineering & ZJU-Hangzhou Global Scientific and Technological Innovation Center, Zhejiang University, Hangzhou 310058, China; ‡ School of Biological Sciences, University of Edinburgh, Edinburgh EH9 3FF, U.K.; § Department of Infection Biology, London School of Hygiene & Tropical Medicine, London WC1E 7HT, U.K.; ∥ School of Life Sciences, University of Westminster, London W1B 2HW, U.K.; ⊥ School of Informatics, University of Edinburgh, Edinburgh EH8 9AB, U.K.; # Research Center for Biological Computation, Zhejiang Laboratory, Hangzhou 311100, China

**Keywords:** *Shigella flexneri*, P1 bacteriophage, CRISPR-Cas9, antimicrobial, phagemid

## Abstract

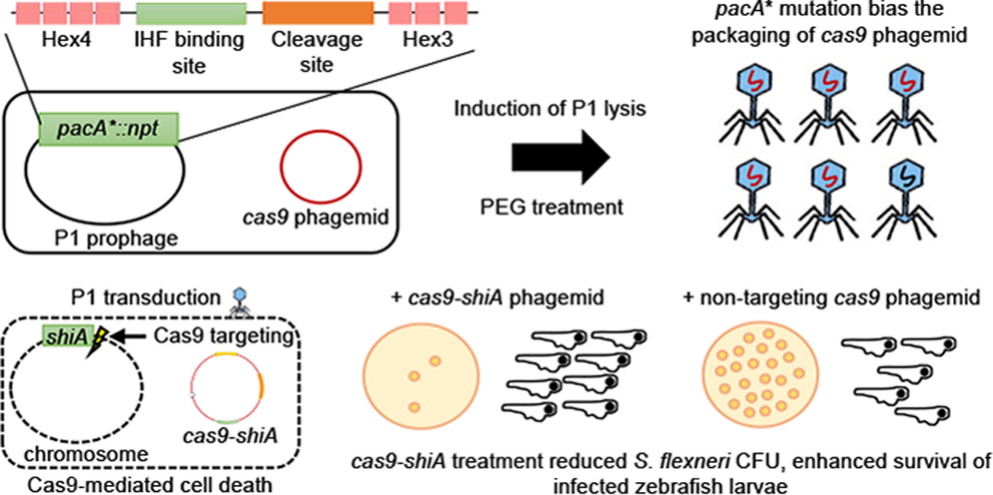

The discovery of clustered, regularly interspaced, short palindromic repeats (CRISPR) and the Cas9 RNA-guided nuclease provides unprecedented opportunities to selectively kill specific populations or species of bacteria. However, the use of CRISPR-Cas9 to clear bacterial infections *in vivo* is hampered by the inefficient delivery of *cas*9 genetic constructs into bacterial cells. Here, we use a broad-host-range P1-derived phagemid to deliver the CRISPR-Cas9 chromosomal-targeting system into *Escherichia coli* and the dysentery-causing *Shigella flexneri* to achieve DNA sequence-specific killing of targeted bacterial cells. We show that genetic modification of the helper P1 phage DNA packaging site (*pac*) significantly enhances the purity of packaged phagemid and improves the Cas9-mediated killing of *S. flexneri* cells. We further demonstrate that P1 phage particles can deliver chromosomal-targeting *cas9* phagemids into *S. flexneriin vivo* using a zebrafish larvae infection model, where they significantly reduce the bacterial load and promote host survival. Our study highlights the potential of combining P1 bacteriophage-based delivery with the CRISPR chromosomal-targeting system to achieve DNA sequence-specific cell lethality and efficient clearance of bacterial infection.

## Introduction

CRISPR (clustered, regularly interspaced, short palindromic repeats), in combination with the Cas (CRISPR-associated) endonuclease enzyme(s), constitutes the immune system of prokaryotes, serving to protect bacteria against invading nucleic acids.^1,2^ Cas9 is an RNA-guided endonuclease that introduces double-strand cleavage at its target DNA sequence, while the specificity is determined by the spacer sequence of CRISPR RNA (crRNA), which is complementary to the target DNA sequence.^3,4^ Previous studies demonstrated that the CRISPR-Cas9 system can be programmed to target antibiotic-resistance gene(s) or species-specific chromosomal gene(s) of bacteria. Such targeting can re-sensitize bacterial populations to antibiotic treatment or, in the latter case, induce cell lethality *via* SOS-mediated responses against double-stranded DNA damage in the bacterial chromosome. Cell lethality *via* DNA sequence-specific targeting has been described for various clinically relevant bacterial pathogens, such as antimicrobial-resistant strains of *Escherichia coli*,^5,6^*Staphylococcus aureus*,^7^ and *Salmonella enterica* spp.^8^ Despite this success, the use of CRISPR-Cas9 as an antimicrobial system is hampered by the low transformation efficiency of target bacteria, especially during infections *in vivo*.^6^

Bacteriophages, viruses that predate bacterial cells, have been used to treat bacterial infections for over a century.^9,10^ New strains of lytic bacteriophages recovered from environmental and biological samples are routinely used in studies to kill clinically relevant and/or multidrug-resistant strains of bacteria, including *Clostridium difficile*,^11^*Shigella flexneri*,^12^*Pseudomonas aeruginosa*,^13^ and methicillin-resistant *S. aureus* (MRSA).^14^ Although the direct use of lytic bacteriophage cocktails is not widely accepted as a reliable alternative to antibiotics in the clinical field, properties of bacteriophages such as their specific host range, high transduction efficiency, stability of bacteriophage particles, and the ability of certain bacteriophages to lysogenize into host cells make them suitable as an efficient delivery tool for genetic constructs.^15,16^ Citorik *et al.* demonstrated M13 phagemid-based delivery of the Cas9 system into carbapenem-resistant *E. coli* to target its antibiotic resistance determinants *bla*_NDM-1_ or *bla*_SHV-18_, which allowed re-sensitization of the bacteria toward antibiotic treatment *in vitro*.^17^ In addition, the Cas9 endonuclease was reprogrammed to target a chromosomally encoded virulence factor (*eae*) of enterohemorrhagic *E. coli*, which allowed DNA sequence-specific killing of the bacteria in a *Galleria mellonella* infection model.^17^ Bikard *et al.* demonstrated the use of the ΦNM1 bacteriophage to deliver phagemid-encoded CRISPR Cas9 antimicrobial systems into *S. aureus*.^5^ In this case, reprograming of the Cas9 endonuclease to target the methicillin resistance gene, *mecA*, was introduced to specifically kill the methicillin-resistant strain USA300Φ but not the RNΦ strain *in vitro*.^5^ The sequence-specific killing effect of Cas9 was expanded to target the chromosomal *aph* gene of the RNKΦ strain, which selectively reduced 40% of the RNKΦ cells *in vivo* using a mouse skin colonization model.^5^ Recently, the M13 phagemid-based delivery of the Cas9 system allowed the selective killing of a F^+^*E. coli* strain in a murine gut colonization model.^18^ In this case, the authors demonstrated a selective reduction of a *gfp*^+^ F^+^*E. coli* strain (approximately 1–3 log), using a *gfp*-targeting M13 *cas*9 phagemid.^18^ Taken together, these studies have suggested that bacteriophages allow efficient delivery of DNA sequence-specific Cas9 antimicrobials into bacteria *in vivo* and strongly support the approach as an alternative therapeutic option to treat bacterial infections.

Shigellosis is an acute intestinal infection caused by *Shigella* spp. Worldwide, it was estimated that *Shigella* caused 80–165 million cases of disease and 600,000 deaths annually.^19^*S. flexneri* is most frequently recorded in developing countries, with a disproportionately high mortality rate in children.^20−22^ Clinical isolates of *S. flexneri* are often drug resistant, and it was estimated that half of all contemporary strains of *Shigella* spp. are multidrug resistant.^23^ This calls for alternative therapeutic options, such as the use of bacteriophages in treating *Shigella* infection. In this study, we demonstrate the use of a broad-host-range transducing P1 phagemid system to deliver chromosomal-targeting *cas9* genetic constructs into *E. coli* and the dysentery-causing *S. flexneri* to achieve sequence-specific lethality of targeted bacterial cells. To reduce the packaging of the resident P1 bacteriophage genome, we performed genetic modification of the *pac* site, the recognition site for the pacase enzyme, to bias the packaging of *cas9* phagemid into P1 phage particles. We show that an improved titer of P1 transducing units in the lysates prepared from pacA*::*npt* EMG16 cells significantly enhanced the Cas9-mediated lethality effect on *S. flexneri*. Moreover, we show that the *cas9* chromosomal-targeting phagemid is efficient in inducing sequence-specific lethality of *S. flexneriin vivo* and significantly improved the survival of infected zebrafish larvae. Overall, these results highlight the potential of P1-based phagemid delivery of the chromosomal-targeting CRISPR-Cas9 system as a powerful tool to target clinically relevant and antibiotic-resistant Gram-negative *Enterobacteriaceae* pathogens.

## Results

### Construction of P1 BBa_J72114 Phagemid with High Transduction Efficiency in *E. coli* Strains

**Figure 1:**
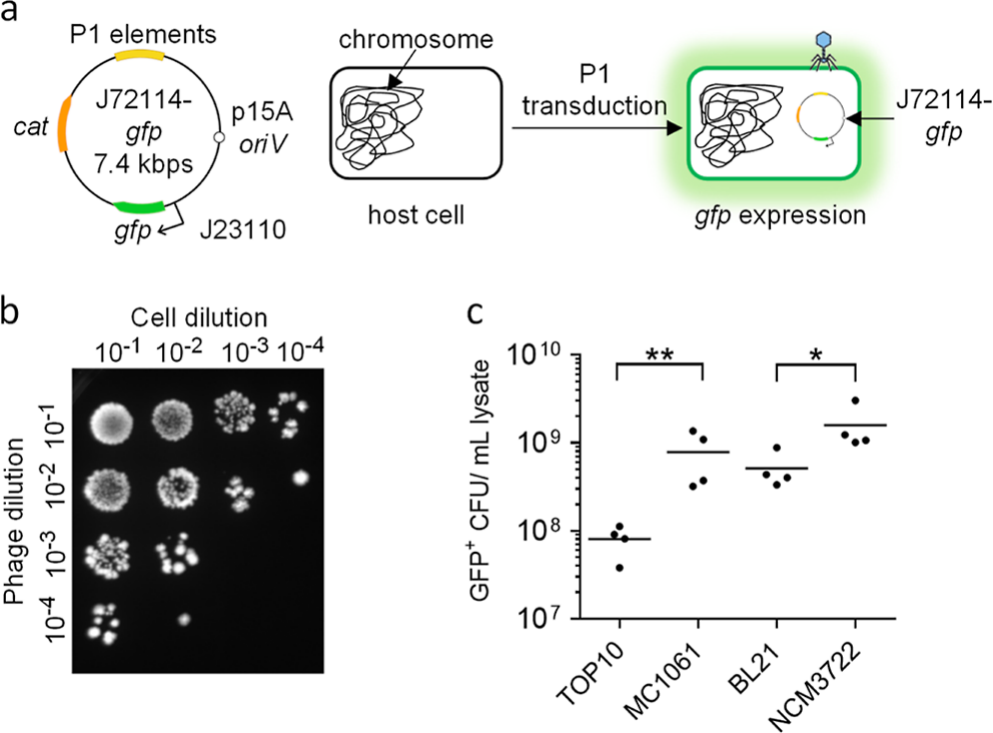
P1 J72114 phagemid as a delivery tool for transduction of a foreign genetic cassette into Gram-negative *Enterobacteriaceae*. (a) Schematic diagram of P1-J72114 phagemid, characterized by constitutive expression of *gfp* placed under the BBa_J23115 promoter. The phagemid contains the chloramphenicol acetyltransferase gene (*cat*), which confers a chloramphenicol-resistant phenotype to transduced or transformed cells. Transduced *E. coli* cells will retain the J72114-*gfp* phagemid, giving constitutive *gfp* expression. (b) Representative image showing the presence of GFP-positive *E. coli* NCM3722 cells after transduction with serially diluted phagemid lysates delivering the *gfp* expression cassette. Serial dilutions of recovered cells (10^1^, 10^2^, 10^3^, and 10^4^) were made and spotted onto LB agar supplemented with chloramphenicol. (c) Quantification of transducing units (phage particles containing phagemid sequences) in various lab strains of *E. coli*. Each data point represents a biological replicate and is the average of four technical repeats. Horizontal lines represent the group mean. The *p*-values were determined and adjusted by the Kruskal–Wallis test and Dunn’s multiple comparison tests, respectively, and significance was defined as *p* < 0.05. * represents *p* < 0.05, while ** represents *p* < 0.005, for comparisons between BL21 *vs* NCM3722 and TOP10 *vs* MC1061 and phagemid transductants, respectively.

To deliver and stably express foreign genetic cassette(s) in *Enterobacteriaceae*, the BBa_J72114 P1 phagemid constructed for this study contains all necessary P1-based elements for packaging of phagemids into P1 bacteriophage particles (refer to Supporting Information Figure S1 for details), as well as a selectable chloramphenicol-resistance marker and p15A or pBBR1 origin of replication for phagemid maintenance (Figure 1a).

Due to the non-replicative nature of the P1 phagemid, we hypothesized that a multiplicity of infection (MOI) greater than or equal to 1 might be required for efficient transduction of the phagemid.^5,17^ To quantify the P1 transducing units’ titer of lysates prepared, the original phagemid was modified to include a constitutive *gfp* expression cassette (J72114-*gfp*, Figure 1a). Various lab strains of *E. coli* were transduced with P1 phage lysates, and recovered cells were selected for chloramphenicol resistance and GFP fluorescence (Figure 1b). The P1 lysates transduced all three substrains of *E. coli* K-12 and *E. coli* BL21 within a range of 7 × 10^8^ to 7 × 10^9^ transducing units per millilitre of lysate used (Figure 1c). Overall, our protocol generates sufficient phagemid titers for the delivery and stable expression of genetic constructs in *E. coli*.

### Efficacy of Cas9-Induced Cell Lethality of *E. coli* MC1061::*npt*

**Figure 2:**
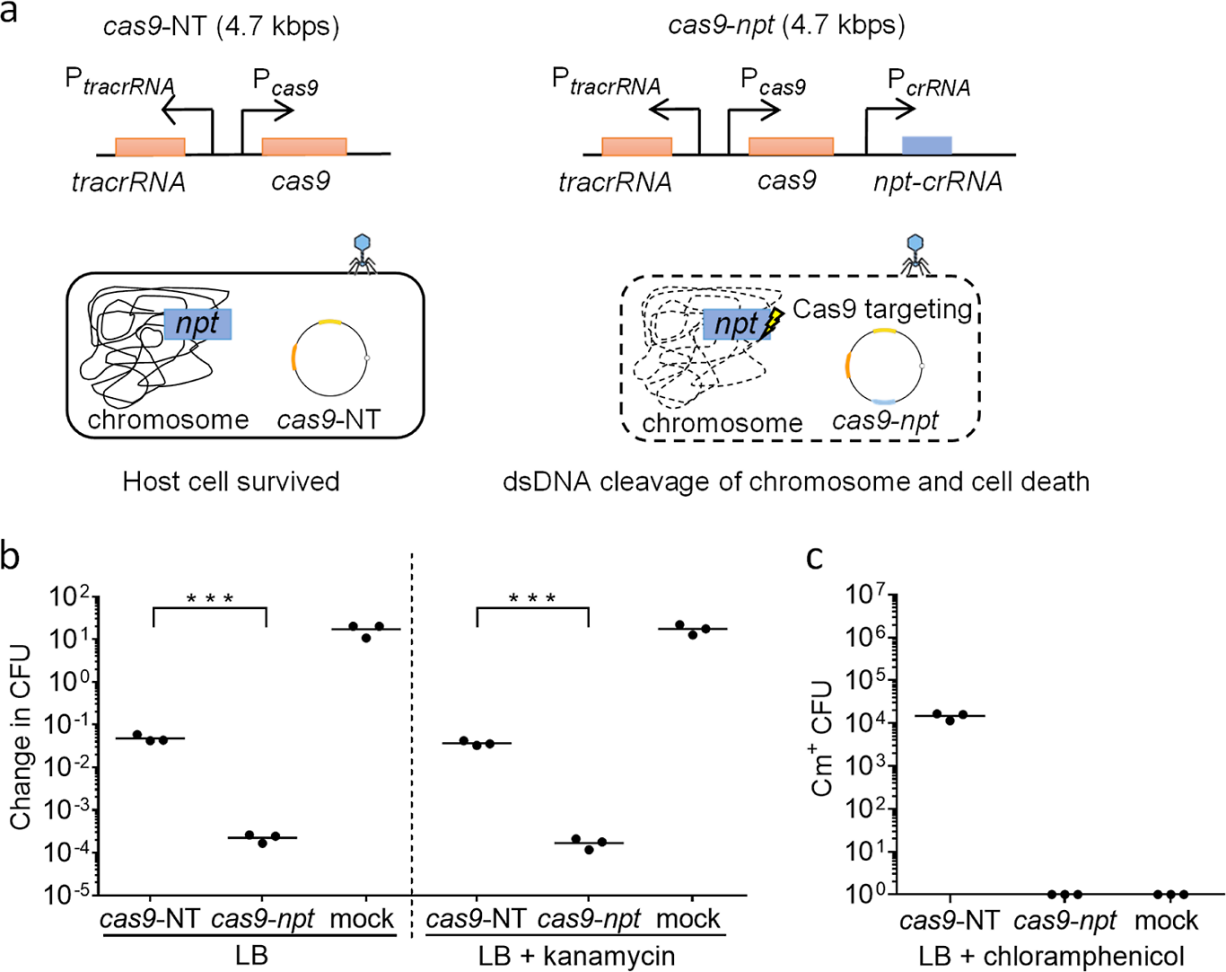
Spacer sequence-mediated lethality of *E. coli* MC1061::*npt* cells using *npt*-targeting *cas9* phagemid. (a) Schematic diagrams showing the *cas9* genetic construct, with or without *npt*-targeting crRNA (*cas9*-*npt* and *cas9*-NT, respectively) assembled onto the P1 J72114 phagemid. The presence of *npt*-targeting *crRNA* would target the Cas9 endonuclease chromosome of *E. coli* MC1061::*npt* cells, causing dsDNA cleavage of the chromosome and cell death. (b) Serial dilutions of transduced *E. coli* MC1061::*npt* were plated onto plain LB agar or LB agar supplemented with kanamycin. Data were plotted as change(s) in CFU as compared to input CFU (approximately 10^7^ cells per reaction) used for infection. (c) Quantification of chloramphenicol-resistant CFUs recovered, after treatment with *cas9*-NT or *cas9*-*npt* phagemid lysates. Each data point represents a biological replicate and is the average of four technical repeats. A MOI of 5 P1 transducing units per bacterial cell was used for all infections. Mock infections involved treating *E. coli* cells with SM buffer. Horizontal bars represent the group mean. The *p*-values (between non-targeting and targeting phagemid treatments) were determined using a Kruskal–Wallis test, with the significance defined by *p* < 0.05. *p* < 0.0005 between targeting phagemid and nontargeting phagemid treatments is shown as ***.

We constructed two J72114-*cas*9 phagemids to achieve sequence-specific DNA cleavage on target enterobacterial cells. The complete Cas9 system was derived from the p*Cas9* plasmid with constitutive expression of *cas9*, *trans*-activating RNA (*tracRNA*), and CRISPR RNA (*crRNA*) (Figure 2a). The specificity and efficacy of Cas9 endonuclease-mediated lethality were first evaluated on the *E. coli* K12 strain MC1061::*npt*, which has a single copy of the chromosomally integrated *npt* gene conferring kanamycin resistance. The *npt*-targeting spacer sequence was cloned into *cas9* phagemid using a BsaI cloning system (refer to Supporting Information Table S3 for DNA sequences). Since *E. coli* MC1061::*npt* is not a recombination-deficient mutant, chromosomal DNA double-strand breaks (DSBs) caused by Cas9 endonuclease(s) in the presence of chromosomal-targeting spacer sequence(s) would induce an SOS-mediated response, leading to DNA repair, cell cycle arrest, and/or apoptosis-like cell death^33^ (Figure 2a). This would lead to a reduced CFU recovery after treatment with *cas9*-*npt* phagemid, as compared to that of *cas9* phagemid without the *npt*-targeting spacer sequence, as well as in mock-infected cells.

The killing effect mediated by the presence of the *npt*-targeting spacer sequence (ΔCFU_NT-*npt*_) was ∼100-fold (*p* < 0.0005) higher than that elicited by nontargeting *cas9*-NT phagemid (Figure 2b). The CFU recovered after treatment with *cas9*-*npt* phagemid was not significantly different in the presence or absence of kanamycin (CFU recovered on plain LB: 65.01 ± 4.97; CFU recovered on LB + kanamycin: 57.14 ± 4.08, *p* > 0.05), suggesting that the reduction in CFU is due to cell lethality caused by Cas9 chromosomal-targeting activity and not to the loss of the *npt* gene and/or its gene function (Figure 2b). These results showed that nontargeting *cas9*-NT phagemid treatment caused ∼25-fold and ∼425-fold reduction in recovered CFU as compared to an input of ∼10^7^ and ∼1.7 × 10^8^ CFU recovered from the mock infection, respectively (Figure 2b). The non-Cas9 spacer sequence-mediated killing effect may be attributed to the general cytotoxic effect of lysates. We did not recover any chloramphenicol-resistant cells after treatment with *cas9-npt* phagemid, suggesting that the presence of both the *npt* gene and the *npt*-targeting spacer sequence of the *cas9* phagemid would always lead to cell death (Figure 2c). There was no significant difference in CFU recovered after *cas9*-*npt* or *cas9*-NT phagemid treatment of *E. coli* K12 MC1061 cells without the chromosomal *npt* gene (Supporting Information Figure S2). This verified that the specificity of Cas9-mediated lethality depends on the presence of both the *npt*-targeting spacer sequence and the chromosomal *npt* gene sequence.

Overall, these results show that the *cas9* phagemid with the chromosomal-targeting spacer sequence is unstable or conditionally lethal when introduced into target bacterial cells.

### Genetic Modification of the *pac* Site of the P1 Genome to Improve P1 Phagemid Purity

**Figure 3:**
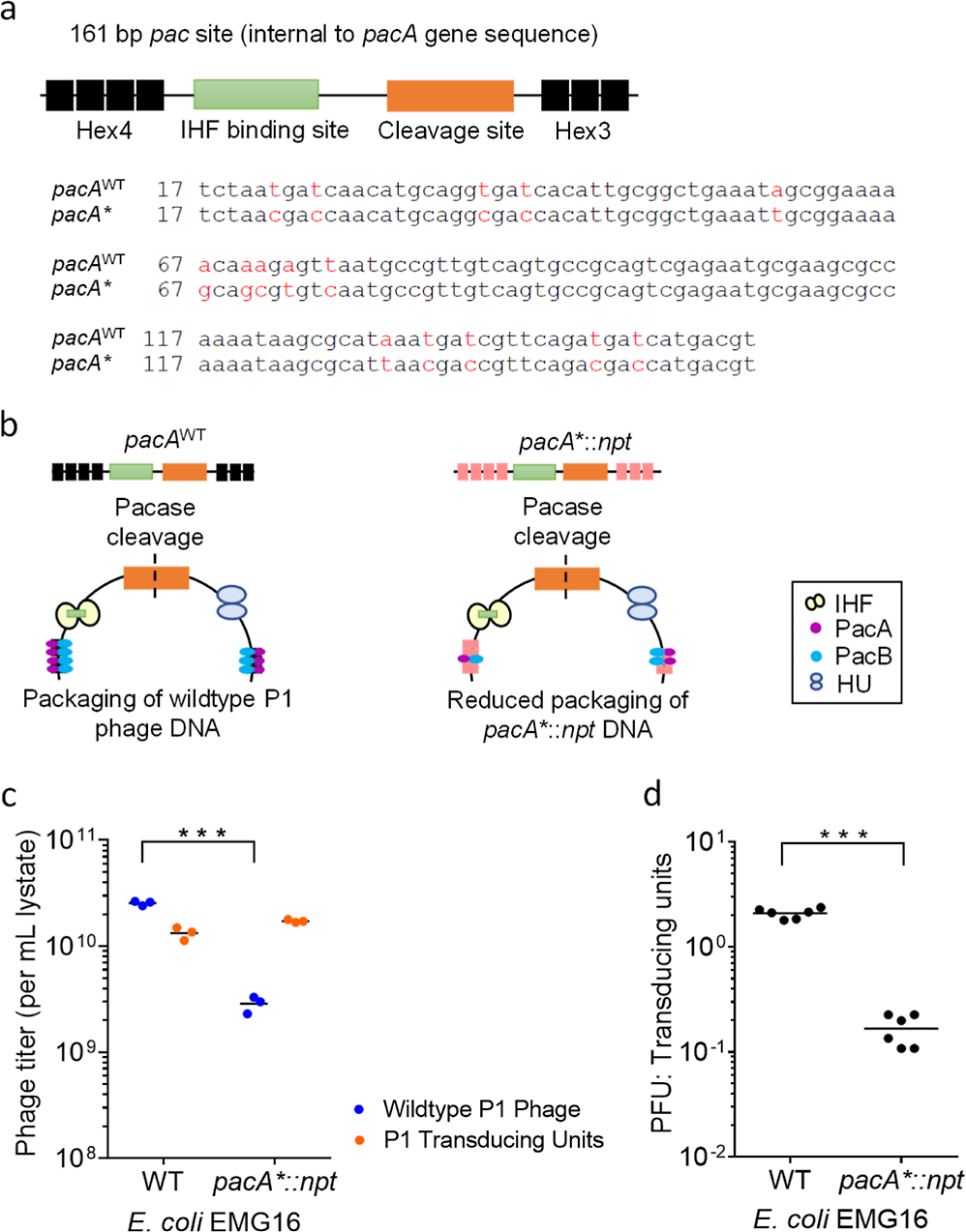
Genetic modification of the *pac* site, within the *pacA* coding sequence of the wildtype P1 bacteriophage genome to reduce wildtype P1 phage DNA packaging. (a) Schematic diagram showing the minimal *pac* sequence of the P1 phage genome, situated within the *pacA* coding sequence. Methylation sites, consisting of hexameric repeats, HEX4 and HEX3, are shown in black, the IHF binding site is shown in green, and the *pac* cleavage site is shown in orange. Synonymous mutations were introduced onto the hexameric repeats to disrupt the binding of PacA and PacB. A Comparison with the wildtype *pac* DNA sequence is shown, with mutated DNA bases indicated in red. (b) Schematic diagram showing the processing of the *pac* site, involving the binding of PacA and PacB to the hexameric repeats, as well as IHF and HU binding, which was proposed to give a bent structure of the *pac* site. Pacase (both PacA and PacB) enzymatic activity then leads to cleavage of the *pac* site.^34,35^ Synonymous mutations introduced onto the hexameric repeats of the *pac* site lead to a reduction in pacase binding, reducing the processing and packaging of the P1 mutant pacA*::*npt* P1 DNA. (c) Quantification of wildtype P1 phage (in blue) and P1 transducing units (in orange) titers of lysates prepared from wildtype (WT) and pacA*::*npt* EMG16 cells harboring J72114 *cas9*-NT phagemid without spacer sequence(s) targeting *E. coli* or *S. flexneri* chromosomal sequences. (d) Ratio of wildtype P1 phage to P1 transducing units was calculated and plotted. Each data point represents a biological replicate and is the average of four technical repeats. Horizontal bars represent the group mean. The *p*-values (between wildtype and pacA*::*npt* lysates) were determined using a two-tailed unpaired *t*-test with significance defined by *p* < 0.05. *p* < 0.0005 between the P1 phage titer of lysates prepared from wildtype and pacA* EMG16 lysogen, as well as the PFU/TU ratio between lysates prepared from wildtype and pacA* EMG16 lysogen are shown as ***.

Previous results showed that P1 phagemid lysates confer general cytotoxicity on *E. coli* K12 MC1061 cells, irrespective of the presence or absence of the Cas9 spacer sequence. The nonspacer sequence-mediated lethality effect (termed general cytotoxicity of lysate) may be attributed to the presence of wildtype P1 phage in the lysates, which are capable of undergoing lytic-stage replication, hence the killing of transduced cells. This prompted us to genetically remove the DNA packaging site, *pac*, on the resident wildtype P1 phage to bias the packaging of cas9 phagemid and reduce wildtype P1 phage titer. The 161 bp *pac* sites lie within the *pacA* gene sequence and contain seven hexameric repeats (“TGATCA/G”) with the “GATC” Dam methylation site.^34,35^ Previous studies proposed that the hemimethylated *pac* site would be recognized and bound by the pacase enzyme, while further methylation would promote cleavage of the *pac* site by the bound pacase.^34−36^ We hypothesized that disruption of these hexamer repeat motifs would reduce the packaging of the wildtype P1 genome. The *E. coli* P1 lysogen EMG16 strain with a modified *pacA* gene sequence (termed pacA*::*npt*) was created by introducing synonymous mutations into the hexameric repeats of the *pac* site *via* lambda-red recombineering (Figure 3a,b). Phage lysates of the *cas9* phagemid without chromosomal-targeting spacer sequences were prepared from wildtype and pacA*::*npt* EMG16 cells. Quantification of plaque-forming units and transducing units suggested that lysates prepared from the pacA*::*npt* mutant contained approximately 9-fold lower wildtype P1 phage titer compared to that of lysates prepared from wildtype EMG16 cells (*p* < 0.0005, Figure 3c). There was no significant difference in the phagemid titers of lysates prepared from both wildtype and pacA*::*npt* EMG16 cells, indicating that the *pacA* genetic modification had negligible effects on the packaging of phagemid into transducing units (*p* > 0.05, Figure 3c).

Overall, these results highlight an improvement in phagemid purity, with a significantly reduced ratio of wildtype P1 phage to phagemid for lysates prepared from the pacA*::*npt* EMG16 mutant cell line (*p* < 0.0005, Figure 3d).

### Efficiency of *cas9* Phagemid-induced Cell Lethality of *S. flexneri*

**Figure 4:**
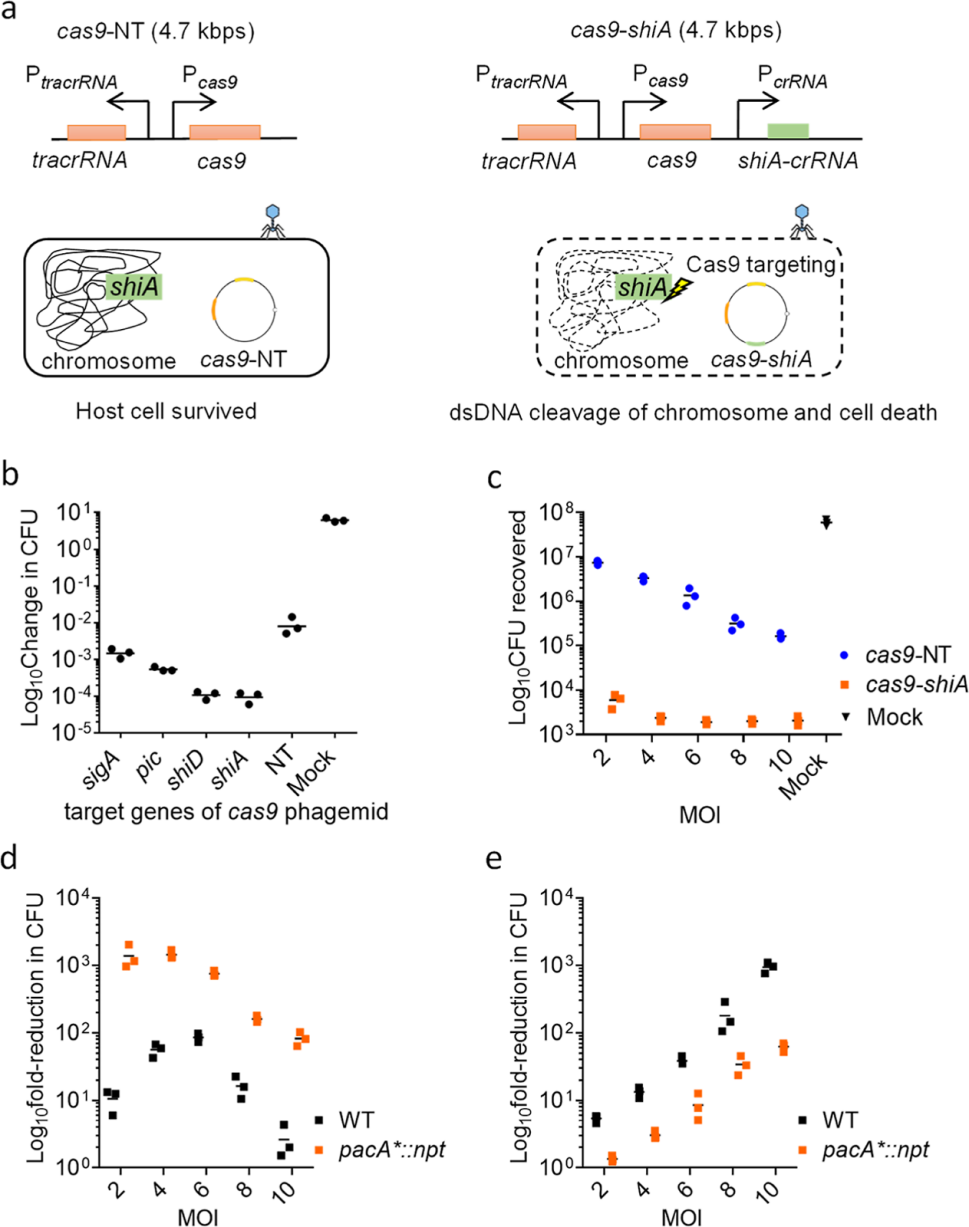
Cas9-mediated lethality of *S. flexneri* using chromosomal-targeting J72114 *cas9*-*shiA* phagemid. (a) Schematic diagram showing the *cas9* genetic construct of P1 J72114 phagemid, with spacer sequence targeting chromosomal gene(s) (i.e., *shiA*) of *S. flexneri* (in green). Upon transduction, the presence of *crRNA* with a spacer sequence complementary to the chromosomal genes of *S. flexneri* (ct-*crRNA*) would cause dsDNA cleavage of the chromosome *via* Cas9 endonuclease, leading to cell death. (b) J72114 *cas9* phagemid with spacer sequence(s) targeting the *sigA*, *pic*, *shiD*, and *shiA* chromosomal genes of *S. flexneri* 2a 2457O. Lysates were prepared from the wildtype EMG16 cell line. Crude lysates were used for transduction of *S. flexneri* 2A 2457O, with a MOI of 10 wildtype P1 phages (equivalent to 5 transducing units) to 1 bacterial cell. Data were plotted as change(s) in CFU as compared to input CFU (approximately 1 × 10^7^ cells per reaction) used for infection. (c) Spacer sequence-mediated lethality of *S. flexneri* 5a MT905 cells with chromosomal-targeting *cas9*-*shiA* (in orange) and nontargeting *cas9*-NT phagemids (in blue) in lysates prepared from the pacA*::*npt* EMG16 cell line. MOIs of 2.0, 4.0, 6.0, 8.0, and 10.0 were used. Data were plotted as changes in CFU as compared to input CFU (approximately 1 × 10^7^ cells per reaction) used for infection. The number of CFU recovered from the mock infection was shown in black. (d) Cas9 spacer sequence-mediated lethality effect of *cas9*-*shiA* phagemid and (e) Nonspacer sequence-mediated lethality effect of *cas9* phagemid lysate prepared from wildtype (WT, in black) and pacA*::*npt* EMG (in orange) cell lines. MOIs of 2, 4, 6, 8, and 10 P1 transducing units to 1 bacterial cell for pacA*::*npt* EMG lysates were used. For wildtype EMG16 lysates, MOIs of 2.0, 4.0, 6.0 , 8.0, and 10.0 wildtype P1 phages per bacterial cell were used, and the wildtype P1 phage to P1 transducing units is approximately 2 for lysates prepared from the wildtype EMG16 cell line. The *cas9*-*shiA*-mediated lethality effect was quantified by measuring the reduction in CFU recovered after treatment with *cas9*-*shiA* and *cas9*-*NT* phagemids. Mock infections involved treating *S. flexneri* cells with SM buffer. The reduction in CFU recovered between *cas9*-NT phagemid treatment and the input cells used for infection (approximately 1 × 10^7^ cells per reaction) would show the nonspacer sequence-mediated lethality effect of P1 phage lysates at all four MOIs tested. Each data point represents a biological repeat and is the average of four technical repeats. Horizontal bars represent the group mean.

Results obtained so far support our hypothesis that the presence of a spacer sequence complementary to an *E. coli* chromosomal gene can lead to cell lethality *via* its Cas9 endonuclease activity. We next chose to test the delivery and properties of the J72114-*cas9* phagemid constructs in the context of *S. flexneri*, the causative agent of shigellosis (also called bacillary dysentery), with high rates of mortality and morbidity among children aged under 5 years in developing countries.^21^ Spacer sequence-mediated lethality effect of the *cas9* phagemid on *S. flexneri* was first tested using *cas9* phagemids with spacer sequences designed to target four conserved virulence (and chromosomal) genes of *S. flexneri*: *sigA*, *pic*, *shiD*, and *shiA* (refer to Supporting Information Table S4 for spacer sequences).^37−40^ To optimize the delivery of the *cas9* phagemid to a new host, a broad-host-range origin of replication, pBBR1, was chosen for the J72114-*cas9* phagemid (Figure 4a). To validate the targeting efficiencies of the spacer sequences designed, we treated an avirulent strain of *S. flexneri* (strain 2a 2457O) using crude P1 *cas9* phagemid lysates prepared from wildtype *E. coli* P1 lysogen. These results showed that spacer sequence(s) targeting the *sigA*, *pic*, *shiD*, and *shiA* genes reduced the number of *S. flexneri* CFU, by ∼5-fold (*p* > 0.05), ∼16-fold (*p* < 0.05), ∼72-fold (*p* < 0.0005), and ∼75-fold (*p* < 0.0005), respectively, when compared to *cas9*-NT phagemid treatment (*p*-values determined with the Kruskal–Wallis test and adjusted with Dunn’s multiple comparison test) (Figure 4b). Since the targeted virulence genes are not linked to the survival of *S. flexneri* cells *in vitro*, the conditional lethality observed is likely to be due to DSB cleavage on the chromosome by the Cas9 endonuclease. These results indicate the functionality of the four spacer sequences in specific targeting of *S. flexneri* chromosomal genes for Cas9-mediated disruption and cell lethality.

We next sought to validate the chromosomal-targeting efficiency of our *cas9*-*shiA* phagemid on a pathogenic strain of *S. flexneri* M90T serotype 5a, which is widely used as a paradigm for cellular microbiology studies and *in vivo* invasion assays.^39^ The *cas9*-*shiA* phagemid was chosen for further transduction assays because it gave the highest spacer sequence-mediated lethality on *S. flexneri* 2a 2457O. P1 lysates of the *cas9*-*shiA* phagemid, as well as its respective non-(chromosomal) targeting constructs (*cas9*-NT), were prepared from pacA*::*npt* EMG16 cells, treated with PEG-6000, and used for transduction assays on *S. flexneri* 5a M90T. To identify the optimal dosage, a range of MOIs (2.0, 4.0, 6.0, 8.0, and 10.0) was used for the transduction assay. These results showed that an MOI value > 2.0 gave the highest spacer sequence-mediated killing effect on *S. flexneri* cells, causing a 3.8 × 10^3^ to 5.1 × 10^3^ reduction in *S. flexneri* CFU (*p* < 0.0005) after treatment with *cas9*-*shiA* phagemid (Figure 4c). The non-Cas9-mediated killing effect at MOIs of 2.0 to 4.0 reduced *S. flexneri* CFU by approximately 3.1-fold (*p* > 0.05) and 6.7-fold (*p* > 0.05), respectively, when compared to an input of 10^7^*S. flexneri* CFU (*p*-values determined with the Kruskal–Wallis test and adjusted with Dunn’s multiple comparison test). We determined that an MOI value of ∼4.0 is optimal, considering that a further increase in MOI would lead to an increase in the non-spacer sequence-mediated lethality effect of lysates without significant changes to the Cas9-mediated lethality effect (Figure 4c). We did not recover any chloramphenicol-resistant *S. flexneri* CFU after treatment with *cas9*-*shiA* phagemid, thus validating the efficiency of its spacer sequence-mediated cell lethality.

To compare the effect of the pacA*::*npt* mutation on improving the quality of lysate, results were compared to those of lysates prepared from wildtype EMG16 cells. Lysates prepared from wildtype EMG16 cells required an MOI of approximately 6.0 for a maximum Cas9-mediated lethality effect of ∼100-fold reduction in CFU (Figure 4d), which was accompanied by a non-spacer sequence-mediated killing effect of approximately 40-fold reduction in CFU (Figure 4e). In contrast, the maximum Cas9-mediated lethality effect of pacA*::*npt* lysates was observed at a MOI of ∼4, producing a ∼16-fold higher DNA sequence-specific killing effect (*p* < 0.05) and a ∼12-fold lower non-Cas9-mediated killing effect (*p* < 0.05), when compared to that of lysates prepared from wildtype P1 lysogen at a MOI of 6 (*p*-values determined with the Kruskal–Wallis test and adjusted with Dunn’s multiple comparison test) (Figure 4d,e).

These results demonstrate the specificity and efficiency of our *cas9*-*shiA* phagemid in killing *S. flexneri* 5a M90T cells *in vitro*. We concluded that pacA*::*npt* lysates could achieve their maximum Cas9-mediated lethality effect at a lower MOI as compared to lysates prepared from wildtype EMG16 cells, hence giving a lower non-spacer sequence killing effect of phagemid lysates on *S. flexneri* cells.

### Validating the Efficiency of the P1 *cas9* Phagemid System *In Vivo* to Control Lethal *S. flexneri* Infection in Zebrafish Larvae

**Figure 5:**
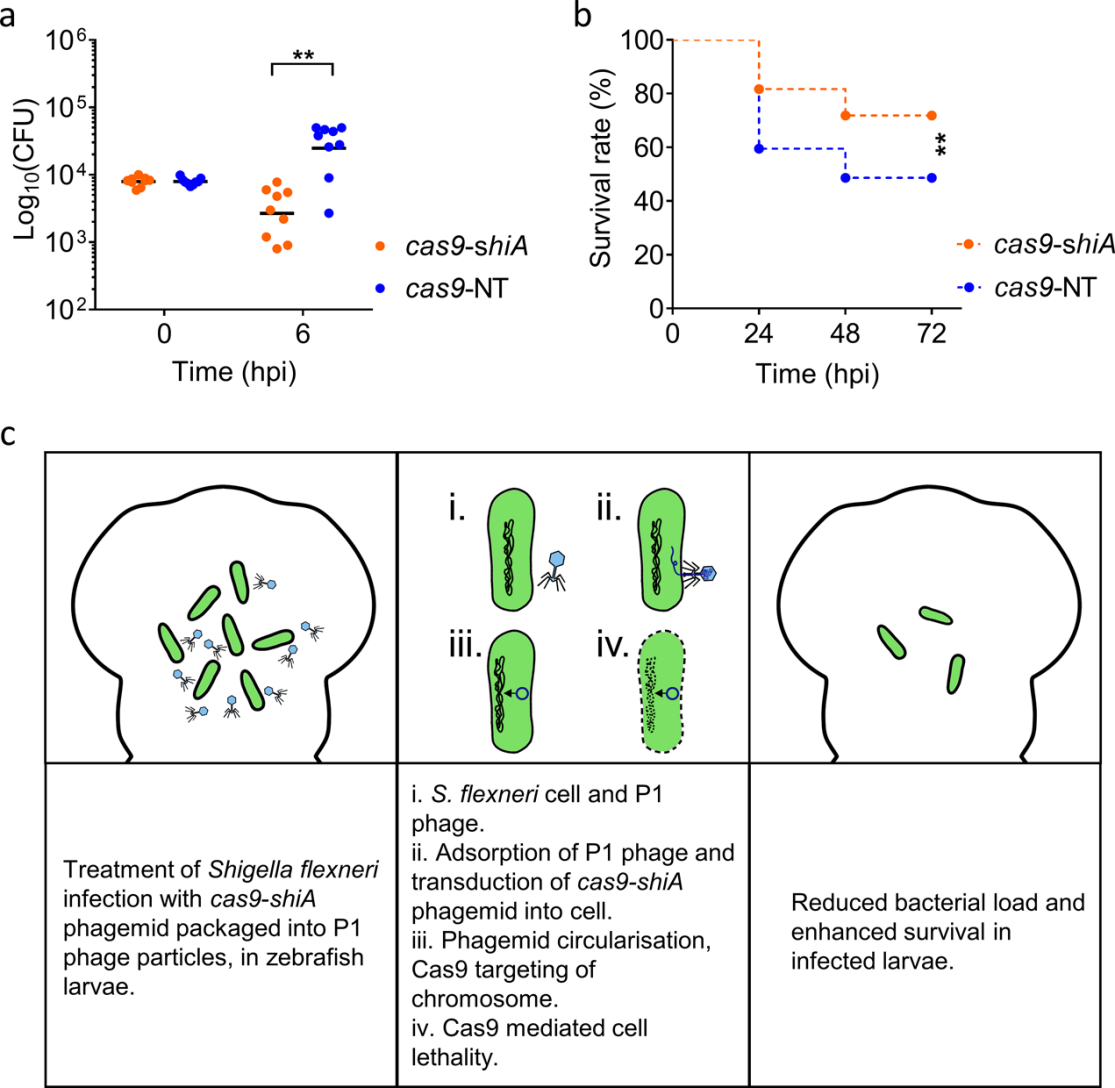
Spacer sequence-mediated lethality of *Shigella flexneri* 5a M90T *in vivo* using chromosomal-targeting *cas9*-*shiA* phagemid. (a) Enumeration of *S. flexneri* 5a M90T CFU at 6 h post-infection. *n* = 9 (targeting); 9 (empty) larvae (cumulated from three independent experiments). (b) Measurement of the survival rate of infected zebrafish larvae at 24, 48, and 72 h post-infection (hpi), treated with *cas9*-*shiA* phagemid (orange) or with the nontargeting *cas9*-NT phagemid (blue). *n* = 71 (targeting); 74 (empty) larvae (cumulated from three independent experiments). Differences in bacterial load were tested using an unpaired *t*-test on log 10-transformed data, while differences in survival were tested using a log-rank (Mantel–Cox) test. *p* < 0.05 is considered statistically significant. *p* < 0.005 between targeting phagemid and nontargeting phagemid treatments are shown as **. (c) Schematic diagram showing the reduction of *S. flexneri* infection bacterial load via the spacer sequence-mediated lethality effect of *cas9*-*shiA* phagemid. The reduced bacterial load would promote the survival of infected larvae.

We next sought to establish whether our P1 *cas9* phagemid system could clear *S. flexneri* infection *in vivo*. A variety of studies have shown that zebrafish larvae are susceptible to *S. flexneri* infection, with key aspects of the human disease being replicated in this model.^41−43^ Zebrafish larvae are recognized as highly versatile for studying innovative treatments against *S. flexneri* infection,^43,44^ for example, clearance of drug-resistant *S. flexneri* infection *in vivo* has been achieved via the injection of the predatory bacteria *Bdellovibrio bacteriovorus*.^42^ To assess the spacer sequence-mediated killing effect of our P1 *cas9* phagemid, P1 phage lysates of *cas9*-*shiA* and *cas9*-NT phagemid (without chromosomal-targeting spacer sequence) were injected into the hindbrain ventricle of zebrafish larvae at 2 days post-fertilization, following the administration of a lethal dose (∼8000 CFU) of *S. flexneri* 5a M90T. We observed an approximately tenfold reduction in *S. flexneri* CFU after treatment with *cas9*-*shiA* phagemid at 6 h post-infection (hpi), compared to that of nontargeting *cas9*-NT phagemid treatment (*p* < 0.005, Figure 5a). This was accompanied by a ∼20% increase in the survival rate of zebrafish larvae (*p* < 0.005) (Figure 5b). In contrast, there were no significant differences in the number of *S. flexneri* CFU recovered at 6 hpi or the survival rate of zebrafish larvae at 24, 48, and 72 hpi between *cas9*-NT phagemid treatment and mock infections (*p* > 0.05) (Supporting Information Figure S3a,b). Injection with either the *cas9*-*shiA* or c*as9*-NT phagemid alone without *S. flexneri* infection did not lead to morphological defects or reduced viability of zebrafish larvae (Supporting Information Figure S4). Overall, these results demonstrate the efficiency of *cas9*-*shiA* chromosomal-targeting phagemid in reducing *S. flexneri* bacterial load *in vivo* and improving the survival of the infected host (Figure 5c). Considering the nonreplicative nature of the P1 phagemid, our results highlight the potential use of *cas9* phagemids as a safe and efficient therapeutic agent *in vivo*.

## Discussion

In this study, we demonstrate P1 phagemid-based delivery of a chromosomal-targeting *cas9* genetic construct into *E. coli* and *S. flexneri* cells. We establish protocols that give a high phagemid titer and introduce the use of pacA*::*npt* EMG16 cell line, which gives improved phagemid purity of lysates. We show efficient killing of *S. flexneri* cells with *cas9* phagemid in the presence of spacer sequences complementary to the chromosomal gene(s) of *S. flexneri*. Finally, treatment of *S. flexneri*-infected zebrafish larvae with chromosomal-targeting *cas9*-*shiA* phagemid significantly reduced the bacterial burden and improved host survival.

We chose the P1 bacteriophage to deliver our phagemids into *E. coli* and *S. flexneri* due to its (1) ability to package large-sized DNA (∼100 kbps), (2) high transduction efficiency among Gram-negative *Enterobacteriaceae*, (3) ability to lysogenize after transduction, and (4) *in trans* induction of single gene expression, *coi*, which can promote lytic stage replication.^25^ Using the P1-phagemid system with arabinose-inducible *coi* gene expression, our results are consistent with observations from previous studies, showing a transduction efficiency of at least 7 × 10^8^ transducing units per mL lysate used on *E. coli*.^24,25^ We observed a significantly lower transduction efficiency of the P1 phagemid for the *E. coli* TOP10 cell, and such reduced transduction efficiency had previously been reported on several *recA*-strains of *E. coli*.^24^ Previous studies suggested that only a subset of P1 phage particles contain Cre recombinase, while the rest of the P1 phage DNA relies on the host cells’ homologous recombination system, such as RecA- and RecBCD-mediated homologous recombination of Chi sites, for the circularization of linear genomic DNA.^45,46^ Chi sites are over-represented in the genome of the P1 bacteriophage, and the *cin* gene sequence contains 2 of the 50 identified Chi sites, which may have improved the transduction efficiency of our P1 phagemid.^46^

Our results demonstrate that the P1 phagemid is efficient in delivering the *cas9* genetic constructs into both *E. coli* and *S. flexneri*. We show that the presence of spacer sequences complementary to the targeted chromosomal gene(s) of *E. coli* and *S. flexneri* yielded a 2 to 3 log reduction in bacterial CFU *in vitro*, which is comparable with the Cas9-chromosomal-targeting effect reported in previous studies.^5,6,17,33^ The efficiency of the *cas9*-*shiA*-targeting phagemid in reducing *S. flexneri* bacterial load *in vivo* was demonstrated as early as 6 hpi, with an approximately tenfold reduction in CFU, thus leading to a ∼20% increase in the survival rate of infected larvae. The reduced *shiA*-targeting spacer sequence-mediated lethality effect, when compared to phagemid treatment *in vitro*, may be due to differences in experimental conditions (i.e., temperature, duration of infection, and environmental conditions), the lifecycle of *S. flexneri* infection *in vivo*, and/or a lower rate of phagemid transduction. In agreement, we did not observe a significant non-Cas9-mediated killing effect of the P1 *cas9* phagemid in zebrafish larvae (when compared to mock infections), so the data suggest a lower rate of phagemid transduction as compared to *in vitro* assays. Our current lysates contain a maximum of ∼4 × 10^9^ to ∼8 × 10^9^ phagemid-transducing particles (per mL sample), which might have limited the rate of phage infection *in vivo*. Furthermore, we hypothesize that the P1 bacteriophage is unable to cross the host epithelial cell membrane efficiently, and thus, the primary target cells of the phagemid might be restricted to the extracellular pool of *S. flexneri*, while intracellular bacteria could proliferate and kill the host. Previous studies showed that repeated administration of phages enhanced clearance of bacterial infections *in vivo*, which might improve the performance of our *cas9* phagemid.^47−49^ However, we did not distinguish whether the surviving *S. flexneri* colonies recovered after *shiA*-targeting phagemid lysate treatment were P1-resistant colonies and/or escape mutants of the Cas9 chromosomal-targeting effect. If the surviving *S. flexneri* are resistant to P1 infection, repeated administration of phagemid lysates may not improve the overall Cas9-mediated killing of bacteria. The factor(s) which may be associated with resistance against phage infection and/or the Cas9 chromosomal-targeting system could be identified by genomic sequencing of surviving *S. flexneri* colonies. These data could provide insights into ways of improving our current P1 phagemid system, such as the engineering of P1 tail fiber to overcome phage resistance, the use of multiple chromosomal-targeting guide RNAs, and/or the use of other RNA-guided endonucleases to enhance the antimicrobial effect. In addition, our study lacks pharmacokinetic analysis to investigate the interactions between P1 phage and the host immune response, which could dictate the efficiency of our P1 phage-based delivery method. Future assessment of the interactions between P1 phage and the host immune system, as well as the concentration of phagemid transducing particles and treatment at higher MOIs, might provide solutions to enhance the stability of P1 phage and rate of phage transduction, which could improve clearance of infection *in vivo*.

Given the nonreplicative nature of the P1 *cas9* phagemid, a higher MOI is required for a significant spacer sequence-mediated lethality effect. However, our results showed that an increase in MOI would also lead to an increase in the general cytotoxicity effect of phagemid lysates on *E. coli* and *S. flexneri*. We demonstrated that this could be mitigated *via* PEG-6000 treatment of lysates and the genetic modification of the *pac* site on the P1 genome by reducing the recovery of wildtype P1 phage and its DNA packaging, respectively. The improved phagemid purity allowed for higher spacer-specific lethality at a lower MOI. It is noteworthy that transduction of a nontargeting *cas9* phagemid leads to a 2 log lower number of *E. coli* and *S. flexneri* CFU when compared to mock infections *in vitro* and the use of PEG-treated lysates prepared on the mutant pacA* lysogen could reduce such non-Cas9-mediated effects on *S. flexneri* by approximately tenfold. While bacterial cells are actively growing in mock infections, the process of P1 phage infection and/or transduction of the *cas9* phagemid could potentially kill and/or reduce the growth rate of bacterial cells. Phage-derived antirestriction proteins, transcription, and the replication of phage DNA are known to trigger various antiphage defense systems, leading to bacterial abortive infection to limit viral infection and replication.^50−53^ Furthermore, given the “headful” DNA packaging mechanism of P1 that requires ∼100 kbp of DNA substrate for phage maturation,^54^ as well as the ability of P1 in packaging DNA substrate without the *pac* sequence,^55^ P1 phagemid transducing particles may be contaminated by bacterial host and/or P1 genomic DNA. Taken together, the presence of P1 antirestriction proteins (i.e., DarA, DarB, and DdrA),^56−59^ cotransduction of host and/or P1 genomic DNA, as well as other aspects of P1 transduction, could potentially contribute toward a lethality effect and/or a lower growth rate of *E. coli* and *S. flexneri*. If this is true, the use of transducing particles containing only the phagemid DNA might eliminate the non-Cas9-mediated killing effect on *S. flexneri* and *E. coli*. Although the well-studied M13-based phagemid system yields a higher titer of pure transducing particles compared to the P1 phagemid system, M13 adsorption requires the tips of F-pili, which restricts its host range to F^+^ cells only.^60,61^ This might limit the efficiency of phagemid delivery, especially in targeting clinical isolates of *S. flexneri*, when compared to P1 phage transduction, which has a broader host range. Tridgett *et al.*, (2021) demonstrated the production of pure cosmid transducing particles, using a mutant P2 lysogen that has its DNA packaging site, *cos*, replaced with P4 δ and ε gene sequences.^62^ However, our previous study indicated a lower transduction efficiency of cosmid DNA into *S. flexneri* 5a M90T by P2 when compared to P1 infection, despite both phages having broad host ranges.^6250^ We are currently developing a P4 cosmid system which could produce pure cosmid transducing particles, using the mutant strain of P2 lysogen described by Tridgett *et al.*(62) Replacing the host range determining region of the P2 tail fiber with that of P1 may improve the transduction efficiency of cosmid DNA into *S. flexneri* 5a M90T. Comparisons between P1 phagemid lysates and P4 cosmid lysates should provide insights into the effects of using pure cosmid transducing particles on the non-Cas9 killing of *S. flexneri*.

The variation across *S. flexneri* serotypes, which differ regionally, is likely to complicate the development of an effective and broadly protective vaccine against *S. flexneri* infection.^63,64^ The versatility of the CRISPR Cas9 system allows seamless reprograming of the endonuclease to target conserved chromosomal DNA sequences and/or virulence factors encoded by *S. flexneri* by modifying its CRISPR guide RNA spacer sequence. Although our results indicate Cas9 killing of *S. flexneri* with four different chromosomal-targeting guide RNAs, a wider panel of spacer sequences may be useful to identify target sites, improving the Cas9-mediated killing of *S. flexneri*. Despite a significant cytotoxic effect of P1 transduction on *S. flexneri*, we demonstrate that P1 transduction of phagemid into both *S. flexneri* serotypes 2a and 5a highlights the great potential of P1 as a universal *S. flexneri* targeting strategy, as the targets can be selected to be conserved across *S. flexneri* serotypes. Furthermore, transduction of phagemid DNA by P1 with its alternative S′ tail fiber is not significantly affected by mutations in the O-antigen modification genes of *S. flexneri* 2a 2457O and 5a M90T.^65^*S. flexneri* serotypes, except serotype 6 (Sf6), share the same O-antigen backbone,^66^ therefore P1(S′) could potentially be exploited to transduce phagemid DNA into other serotypes of *S. flexneri*. We also assessed P1(S′) transduction on serotype 2b, in which preliminary results suggested a higher number of phagemid transductants when compared to P1(S) infection.^65^ In the future, it will be interesting to assess P1 transduction efficiency on the remaining *S. flexneri* serotypes to determine if the P1 *cas9* phagemid can provide broad and efficient targeting of the bacteria.

In summary, combining CRISPR-Cas9 sequence-specific bacterial targeting with P1 bacteriophage-based delivery has great potential to be used as a supplement to conventional antibiotics for the treatment of antibiotic-resistant bacterial infections. As demonstrated in this study, the genetic modification of P1 bacteriophage and the incorporation of the CRISPR-Cas9 system in the form of phagemid are useful for targeting clinically relevant Gram-negative *Enterobacteriaceae*.

## Materials and Methods

### Bacterial and Phage Strains, Plasmids/Phagemids, and Growth Media

The strains of *E. coli* and *S. flexneri* used for this study are listed in Supporting Information Table S1, along with a full description of strain modification (if any) and the purpose of each strain used in this study. Plasmids/phagemids used in this study are listed in Supporting Information Table S2 and were constructed using Gibson assembly. DNA sequences of constructs are listed in Supporting Information Table S3. Bacterial cells were cultured in Luria–Bertani medium (LB) or phage lysis medium (PLM; LB containing 100 mM MgCl_2_ and 5 mM CaCl_2_), while SM buffer (50 mM Tris–HCl, 8 mM MgSO_4_, and 100 mM NaCl, pH 7.5) was used for P1 bacteriophage manipulation, as stated in previous studies of P1 bacteriophage.^24,25^ Concentrations of antibiotics used were 50 μg mL^–1^ for ampicillin and kanamycin and 25 μg mL^–1^ for chloramphenicol. All chemical reagents used were analytical grade and purchased from Sigma-Aldrich.

### Bsa1 Cloning of Chromosomal-Targeting Protospacer Sequences

The CRISPR Cas9 construct used in this study was derived from a pCas9 plasmid (Addgene plasmid #42876), which contain the *cas9* gene under a constitutive promoter, a *trans*-activating CRISPR RNA (*tracrRNA*) and a CRISPR guide RNA (*crRNA*). The *crRNA* sequence contains two BsaI sites that allow molecular cloning of a protospacer sequence. 20 bps protospacer sequences targeting the *npt* and *S. flexneri* chromosomal sequence(s) with an NGG protospacer adjacent motif (PAM) were designed using the CHOP–CHOP web tool.^26^ Primer pairs having a protospacer sequence were designed to have compatible ends for its annealing into the Bsa1-digested sites of the *crRNA* sequence. The design, annealing, and cloning of primer pairs into the *crRNA* sequence were performed as described by Jiang *et al.*, (2013).^27^ Restriction digestion of phagemid DNA was carried out using BsaI-HFv2 (NEB), following the manufacturer’s protocol. Primer pairs used for protospacer sequence cloning are listed in Supporting Information Table S4.

### Phage Lysate Preparation

Phage lysates were prepared using a previously established protocol, with some modifications.^25^ Briefly, *E. coli* EMG16 harboring the P1_kc_ lysogen (P1 is used throughout the text instead) were chemically transformed with the J72114-*cas9* phagemids using a standard protocol for heat-shocked transformation of *E. coli* cells. An overnight culture of transformed *E. coli* P1 lysogen was diluted 1/100 in fresh PLM media and cultured for 1 h at 37 °C. Cell lysis was induced *via* the addition of l-arabinose (final concentration of 13 mM). Cell lysis was defined as the presence of debris and the clearance of bacterial culture, which happened at approximately 2 h post-induction with 13 mM l-arabinose. Chloroform (final concentration of 2.5%) was added to lysates, and cultures were shaken for 30 min to aid in thorough cell lysis and lysate sterilization. Cultures were vortexed, lysates were clarified *via* centrifugation at maximum speed (16,000*g* for 3 min), and the supernatant was collected. The supernatant was then passed through a 0.22 μm syringe filter (Millipore) to remove the remaining cell debris. At this stage, the lysates prepared were identified as “crude lysate” and stored at 4 °C.

Our preliminary results suggested that lysates produced from *E. coli* strain NCM3722 harboring P1 contained a higher number of transducing units as compared to that of strain EMG16 (Supporting Information Figure S5). For the preparation of phage lysates using NCM3722 P1, an overnight culture of *E. coli* NCM3722 cells was diluted in fresh LB medium and cultured at 37 °C until an OD_600_ of approximately 1.0. Cells were concentrated tenfold in PLM medium. Crude lysates prepared from *E. coli* EMG16 cells were used to transduce NCM3722 cells (refer to the transduction section of Materials and Methods). Cells were plated onto LB agar with kanamycin and chloramphenicol, which select for both the pacA*::*npt* P1 and the J72114-phagemids, respectively, and plates were incubated at 37 °C for at least 16 h. For lysates prepared from wildtype P1 lysogen, transduced NCM3722 cells were plated onto LB agar with chloramphenicol only. Colonies were picked, and PCR reaction(s) were carried out using primers that anneal specifically to genes of the P1 genome, such as *lpa*. The same protocol for arabinose induction of cell lysis mentioned above was used for making lysates from NCM3722 cells harboring both P1 and the J72114-phagemids.

### PEG-6000 Treatment of Phage Lysates

Treatment of crude lysates with PEG-6000 was carried out based on protocols established in previous studies but with slight modification.^28,29^ Briefly, the crude lysates prepared were first treated with NaCl to a final concentration of 0.33 M and incubated on ice for 1 h. This step was taken to ensure the precipitation of debris and proteins in the lysates. Lysates were centrifuged at 5000*g* for 50 min, and the supernatant was collected and further treated with PEG-6000 to a final concentration of 4%, at 4 °C overnight. The use of 4% PEG-6000 was justified by our results, which showed a significant reduction in the amount of wildtype P1 phage and a substantial number of transducing units recovered from the lysates (data not shown). After overnight incubation with PEG-6000, the phage precipitate was spun down at 5000*g* for 1 h at 4 °C. The supernatant was removed, and the phage precipitate was resuspended in an appropriate volume of SM buffer; that is, 50× concentration of the phage particle would require resuspending the phage precipitate with 1 mL of SM buffer for 50 mL of lysate. Lysates were filtered through a 0.22 μm syringe filter (Millipore) and further washed or concentrated using Amicon Ultra MWCO 100 kDa centrifugal filter units (Millipore). Lysates were then stored at 4 °C.

### Quantification of Plaque-Forming Units

Plaque assay was carried out to estimate the population of wildtype P1 phage in lysates, using previously established protocols.^30^ Briefly, a stationary phase culture of the naïve *E. coli* host strain, NCM3722, was diluted in fresh PLM broth by 1/100 and cultured at 37 °C with shaking until an OD_600_ of 0.5, which took approximately 2.5 h. From our preliminary results, phage lysates prepared from wildtype and pacA*::*npt* P1 *E. coli* lysogen would have to be diluted to 10^7^ and 10^6^, respectively, to give a reasonable amount (50 to 200) of PFU. 1 mL of cell suspension was added to 100 μL of diluted phage lysate, vortexed, and incubated at 37 °C with shaking for 10 min. Cells and phage mixture were added to 3 mL of melted top agar (LB medium with 0.6% agar) and then poured immediately onto bottom agar (LB medium with 1.5% agar). Agar plates were dried at room temperature for 10 min and then incubated at 37 °C for at least 16 h before the enumeration of PFU.

### Quantification of Transducing Units/Phagemid

The transduction assay was carried out based on the protocol established in a previous study with slight modifications.^25^ Instead of using a stationary phase culture for transduction, an overnight culture of the indicator *E. coli* strain NCM3722 was first diluted in fresh LB broth by 1/100 and cultured at 37 °C with shaking until an OD_600_ of 0.5. Cells were spun down at 3000*g* for 5 min and concentrated tenfold in fresh PLM buffer, providing approximately 10^8^ cells per 100 μL of bacterial suspension for transduction. An equal volume of diluted (10^1^ to 10^2^ dilution factor) phage lysate was mixed with the resuspended cells, and phage adsorption was allowed for a maximum of 30 min at 37 °C with shaking. SOC with 10 mM sodium citrate was added to the cell and phage lysate mixture for cell recovery, expression of an antibiotic resistance marker, and quenching of further phage infection *via* citrate interaction with free calcium ions needed for phage adsorption. SOC recovery was carried out at 37 °C for 1 h with shaking. Serial dilutions of recovered cells were performed and spotted onto plain LB agar as well as LB agar supplemented with 25 μg/mL of chloramphenicol. Agar plates were incubated at 37 °C for at least 16 h before the enumeration of chloramphenicol-resistant colonies. Transducing efficiency would be defined by the percentage of chloramphenicol-resistant colonies against the total CFU recovered on plain LB agar.

### *pacA* Genetic Modification

The lambda red recombineering technique was used for genetic manipulation of the *pacA* gene of the P1 genome. The template for recombination was designed to have homology arms complementary to the 3′-end of *lpa* gene and the 5′-end of *pac* sequence (Supporting Information Tables S3). Synonymous mutations were introduced via codon optimization of the *pac* site to disrupt the hexameric repeats. A kanamycin resistance cassette was included in the *pacA* modification template for lambda red recombination as a selection marker for positive clones, which would be integrated into the intergenic region between the *lpa* gene and the Lp_Pac_ promoter. The *pacA* modification template was first cloned into an empty plasmid with pSC101 as the origin of replication *via* Gibson assembly, and sequenced and verified. The linear dsDNA substrate used for lambda red recombination was produced *via* PCR, followed by gel extraction of the PCR product. Lambda red recombineering was carried out based on previously established protocol.^31^ Briefly, wildtype *E. coli* EMG16 P1 lysogen was transformed with the pKD46 plasmid. Stationary phase culture of the transformed cells was grown in fresh LB at a dilution factor of 100, at 30 °C with shaking, until an OD_600_ of 0.35. 0.65 M l-arabinose was added to the culture to induce the expression of lambda Red genes (*exo*, *bet*, *gam*) and cultured for a maximum of 30 min at 30 °C, with shaking. Cells were chilled on ice for 40 min and made electrocompetent using standard protocols for preparing electrocompetent cells. 100 ng of dsDNA substrate was electroporated into the cells, followed by growth in SOC at 30 °C with shaking for 2 h. Cells were plated onto LB agar supplemented with 50 μg/mL of kanamycin, and incubated at 37 °C for at least 16 h. Colony PCR was carried out on the colonies recovered, using primer pairs that (a) anneal to the junction of integration to verify the correct insertion of the modification template (refer to Supporting Information Figure S6) and (b) are complementary to the modified nucleotide bases to select for colonies that retain the mutations to the *pac* site. PCR products (using primers annealing to the junction of integration) of the correct size were excised and sequence-verified. Positive clones were re-streaked onto new LB agar supplemented with 50 μg/mL of kanamycin, incubated at 37 °C for at least 16 h; this re-streaking process was repeated for at least three generations to ensure homogeneity in the bacterial colony. The kanamycin resistance cassette was retained in the mutant cell line, which provided a selectable marker for the pacA*::*npt* P1 lysogen.

### *E. coli* MC1061::*npt* and *S. flexneri* Chromosomal-Targeting Assay

Stationary phase culture of naïve host cells *E. coli* MC1061::*npt* were sub-cultured in fresh PLM at a dilution factor of 100 at 37 °C until an OD_600_ of 0.35 is reached. Cells were then diluted to reach an OD_600_ of 0.1 in fresh PLM, which gave approximately 1 × 10^8^ cells per mL culture. 100 μL of the diluted culture, giving 1 × 10^7^ cells, was mixed with an equal volume of phage lysate diluted to the intended MOI. Phage adsorption was allowed for 30 min with shaking at 37 °C. Cells were then recovered, and further phage infection was quenched by the addition of SOC with 10 mM sodium citrate for 1 h at 37 °C with shaking. Serial dilutions of cells were made and spotted onto plain LB agar and/or LB agar with 25 μg/mL of chloramphenicol or 50 μg/mL of kanamycin. To enumerate the input used for the chromosomal-targeting assay, 100 μL of the diluted culture was combined with an equal volume of SM buffer, followed by the addition of SOC with 10 mM sodium citrate, and then plated onto plain LB agar and/or LB agar with 50 μg/mL of kanamycin. Mock-infected cells were treated the same as input cells for phage lysate treatment, but with 100 μL of SM buffer as a negative control. Agar plates were incubated at 37 °C for at least 16 h before enumeration of CFU. The number of recovered CFU was then normalised to that of input cells, except for those plated on LB agar with chloramphenicol, whereby data was normalized to the number of CFU recovered after treatment with phagemids without *npt*-targeting spacer sequence, since input cells would not have the phagemid hence not chloramphenicol resistant.

### Zebrafish Larvae Model for *In VivoS. flexneri* Infection

Animal experiments were performed according to the Animals (Scientific Procedures) Act 1986 and approved by the Home Office (Project license: P4E664E3C). Protocols are in compliance with standard procedures as reported at zfin.org. Unless specified otherwise, eggs, embryos, and larvae were reared at 28.5 °C in 0.5 × 10^2^ medium supplemented with 0.3 μg/mL methylene blue. Injections were performed under anesthesia, obtained by supplementing the medium with buffered 200 μg/mL tricaine.

GFP fluorescent and carbenicillin-resistant *S. flexneri* 5a M90T was prepared for injections as in Torraca *et al.* 2019.^32^ Bacteria were suspended to ∼8000 CFU/nL in PBS containing 2% polyvinylpyrrolidone and 0.5% phenol red. 1 nL of the bacterial suspension was microinjected in the hindbrain ventricle of 2 days post-fertilization (dpf) zebrafish larvae. At 45 min post-infection, infected larvae were injected with a phagemid suspension (3 nL of a phagemid solution in SM Buffer + 5 mM CaCl_2_, corresponding to ∼10^4^ P1 transducing units).

Following phagemid delivery, larvae were incubated at 32.5 °C. The survival rate was recorded at 24, 48, and 72 h post-infection. Bacterial enumeration from zebrafish was performed at 0 and 6 hpi by mechanical disruption of infected larvae in 0.4% Triton X-100 and plating of serial dilutions onto Congo red-tryptic soy agar plates containing 100 μg/mL carbenicillin.

### Statistical Analysis

All experiments were carried out with at least three biological and four technical repeats. Calculations of results were performed in Excel (Microsoft, Redmond, WA, USA). GraphPad Prism 6 was used to generate graphs and for statistical analysis. A Shapiro–Wilk normality test was used to determine the distribution of data. To determine statistical significance, Student’s *t*-test (unequal variance, 2-tailed) or the Kruskal–Wallis test was carried out for the normally distributed and non-normally distributed data sets, respectively. For comparisons involving multiple groups, a Dunn’s multiple comparison test was used to adjust the *p*-values determined by the Kruskal–Wallis test. For zebrafish experiments, differences in bacterial load were tested using an unpaired *t*-test on log 10-transformed data (Figure 5a, Supporting Information Figure S3a), while differences in survival were tested using a log-rank (Mantel–Cox) test (Figure 5b, Supporting Information Figure S3b). The statistical test for each of the data sets was listed in the respective figure legend as well as in the main text. Data are expressed as means. *p* < 0.05 is considered statistically significant. Stars on graphs represent *p*-values for statistically significant comparisons, with * representing *p* < 0.05, ** representing *p* < 0.005, *** representing *p* < 0.0005, and n.s. representing *p* > 0.05.

## Supporting Information Available

Phagemid construct design; specificity of Cas9 chromosomal-targeting activity; measurement of the non-Cas9-mediated lethality effect of *S. flexneri* in zebrafish larvae; viability of zebrafish larvae after lysate treatment; comparison of phagemid titers between NCM3722 and EMG16 lysogens; DNA gel verifying the *pacA**::*npt* mutation; relevant genotypes of bacterial strains used; plasmids and phagemids used; primers used; and DNA sequences for all plasmids and phagemids used in this study (PDF)

## Notes

All data in the main text and the Supporting Information are available from the corresponding author upon reasonable request.
