# Supplementary material for: P1 Bacteriophage-Enabled Delivery of CRISPR-Cas9 Antimicrobial Activity Against Shigella flexneri
Source: ACS Synth Biol. 2023 Feb 20;12(3):709–21. doi: 10.1021/acssynbio.2c00465 (PMC10028697; doi:10.1021/acssynbio.2c00465)
Supplement: Supplementary file 1 — sb2c00465_si_001.pdf [file sb2c00465_si_001.pdf]

Supplementary Information for

"P1 bacteriophage-enabled delivery of CRISPR-Cas9 antimicrobial activity against *Shigella flexneri*"

Yang W. Huan[2#], Vincenzo Torraca[3,4#], Russell Brown[2#], Jidapha Fa-arun[2], Sydney L. Miles[3], Diego A. Oyarzún[2,5], Serge Mostowy[3]* & Baojun Wang[1,6]*

[1]College of Chemical and Biological Engineering & ZJU-Hangzhou Global Scientific and Technological Innovation Center, Zhejiang University, Hangzhou 310058, China

[2]School of Biological Sciences, University of Edinburgh, Edinburgh EH9 3FF, United Kingdom

[3]Department of Infection Biology, London School of Hygiene & Tropical Medicine, London WC1E 7HT, United Kingdom

[4]School of Life Sciences, University of Westminster, London W1B 2HW, United Kingdom

[5]School of Informatics, University of Edinburgh, Edinburgh EH8 9AB, United Kingdom

[6]Research Center for Biological Computation, Zhejiang Laboratory, Hangzhou 311100, China

*Corresponding authors (Baojun Wang: baojun.wang@zju.edu.cn; Serge Mostowy: serge.mostowy@lshtm.ac.uk)

[#]Equal contribution

Table of Contents

Supplementary Figures 1-6

Supplementary Tables 1-4

Supplementary References

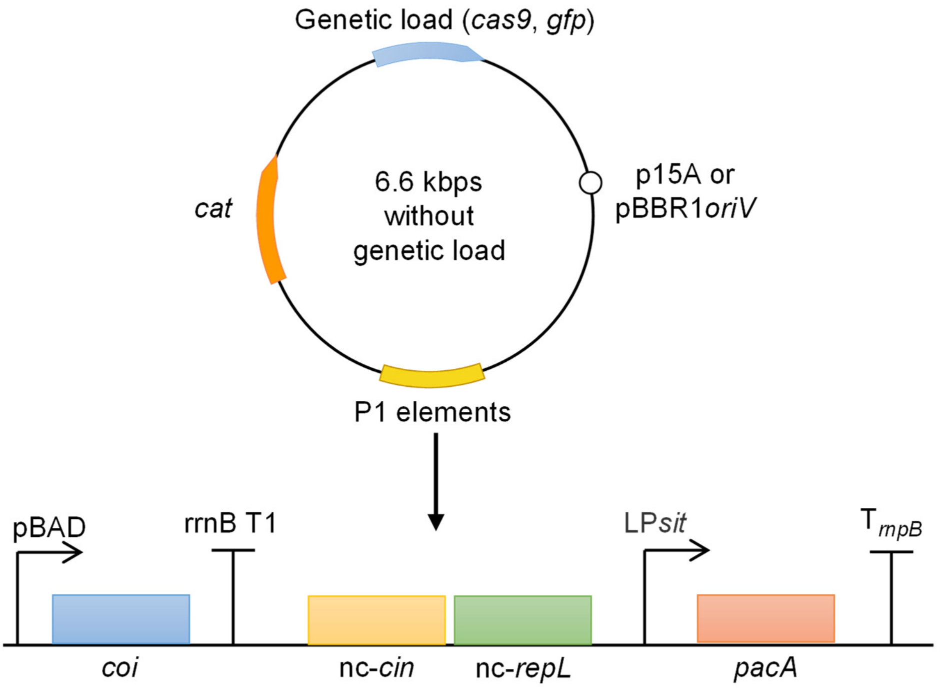

Supplementary Figure 1: Schematic diagram showing the design of the J72114 phagemid. The P1-based elements of our J72114 phagemid series are *coi*, *pacA*, non-coding copy of *repL* and *cin* gene sequences[1,2]. *coi*, whose gene product acts as a repressor antagonist of c1 repressor, hence acting as the switch that induces the lytic stage replication of P1 bacteriophage[3]. The *coi* gene expression of the BBa_J72114 phagemid was placed under the regulation of arabinose-inducible promoter, P_{BAD}, thus allowing *trans*-activation of the phagemid packaging into phage particles in the presence of arabinose. A non-coding copy of the P1 *repL* gene, which contains the P1 lytic stage origin of replication, ori_L, was included in the BBa_J72114 phagemid, allowing the lytic replicase generating phagemid DNA in the linear concatenated form required for packaging into the P1 virion during virus assembly[1,2]. A copy of the P1 *pacA* gene is also included under the control of the late lytic promoter LP_{sit}. The hexamer repeat motif of *pacA* is recognised by the P1 pacase, which cleaves at this site and brings the concatemeric DNA into an empty P1 capsid for packaging[1,2]. Finally, a non-coding copy of *cin*, a recombinase involved in expanding the P1 host range through tail-fibre variation, is included to increase phagemid packaging efficiency via an unknown mechanism[2]. The p15a origin of replication was swapped to the broader host spectrum pBBR1 for the phagemids used for chromosomal targeting of *S. flexneri.* All of our J72114 phagemid series contain a copy of *cat* gene, which confers transduced bacterial cells with chloramphenicol resistance.

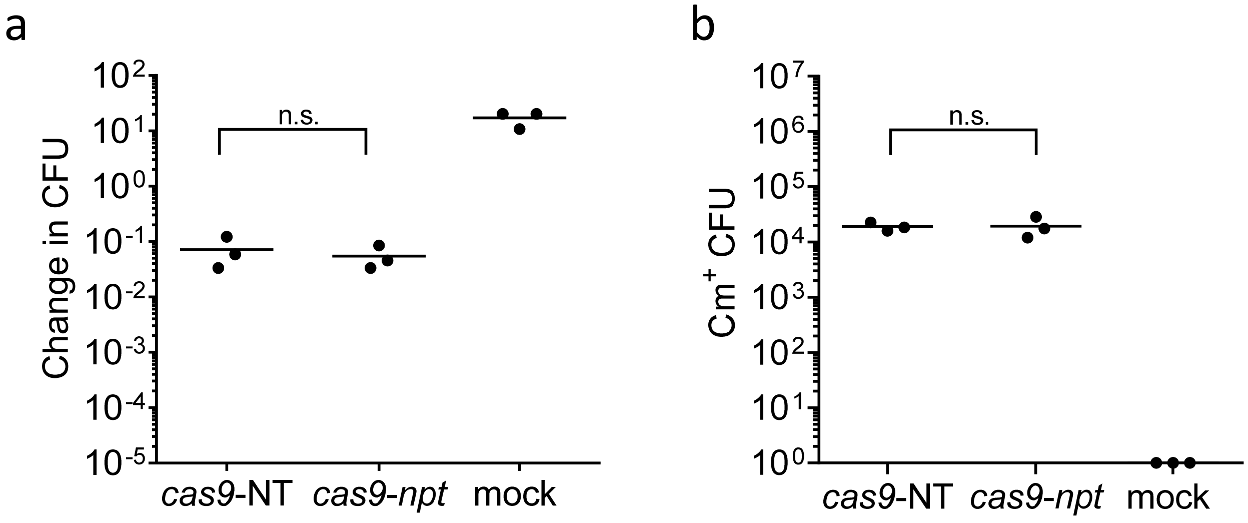

Supplementary Figure 2: No significant Cas9-mediated lethality effect in *E. coli* K12 MC1061 cells without the chromosomal *npt* gene. (**a**) Serial dilutions of transduced *E. coli* K12 MC1061 cells were plated onto plain LB agar. Data were plotted as change(s) in CFU as compared to input CFU (approximately 10^7 cells per reaction) used for infection. (**b**) Quantification of chloramphenicol-resistant CFUs recovered, after treatment with *cas9*-NT or *cas9-npt* phagemid lysates. Each data point represents a biological replicate and is the average of 4 technical repeats. A multiplicity of infection (MOI) of 10 wildtype P1 phage (equivalent to 5 transducing units) to 1 bacterial cell was used for all infections. Mock infections involved treating *E. coli* cells with SM buffer. Data was represented in the form of mean. The p-values (between non-targeting and targeting phagemid treatments) were determined using a two-tailed unpaired t-test with significance defined by $p < 0.05$. n.s. represents no significant difference(s) in the changes in CFU, as well as the number of chloramphenicol-resistant CFU (representing phagemid transductants) between non-targeting and *npt*-targeting phagemid treatment of *E. coli* MC1061.

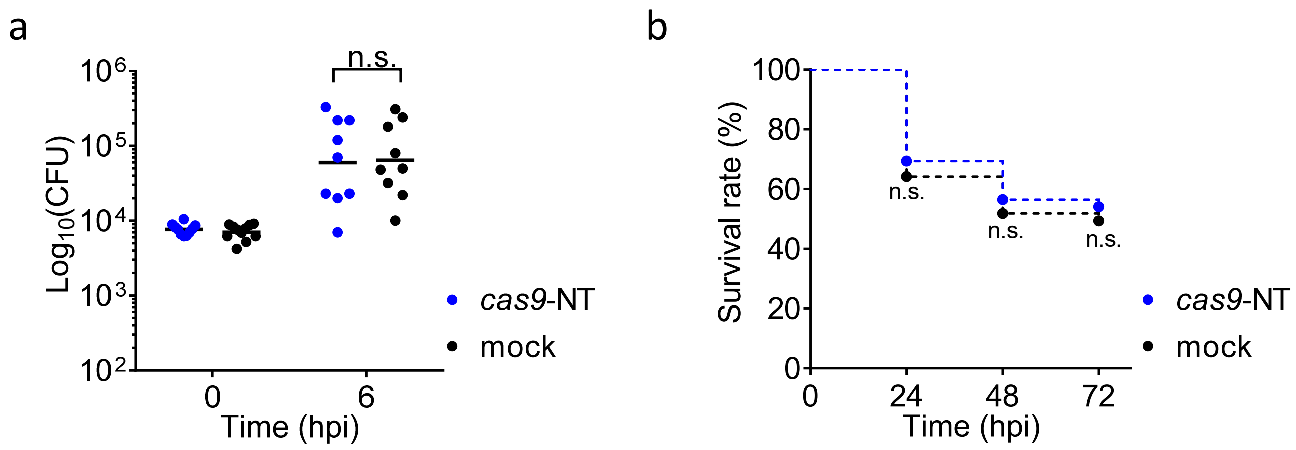

Supplementary Figure 3: No significant differences in the *S. flexneri* load and survival rate of zebrafish larvae between mock infection and after *cas9*-NT phagemid treatment. (**a**) Enumeration of *S. flexneri* 5a M90T CFU at 6 h post-infection after *cas9*-NT phagemid treatment (blue). Mock infection (black) involves treating zebrafish larvae with equal volume (3 nL) of SM Buffer + 5 mM $CaCl_2$, n = 9 to 10 (*cas9*-NT, empty); 9 to 11 (mock infection) larvae (cumulated from 3 independent experiments). (**b**) Measurement of survival rate of infected zebrafish larvae at 24, 48 and 72 h post-infection (hpi), treated with *cas9*-NT phagemid (blue) and mock infection (black). n = 61 (*cas9*-NT, empty); 57 (mock infection) larvae (cumulated from 3 independent experiments). Non-significant (n.s.) differences in bacterial load were tested using an unpaired t-test on Log10-transformed data while differences in survival were tested using a Log-rank (Mantel-Cox) test ($p > 0.05$).

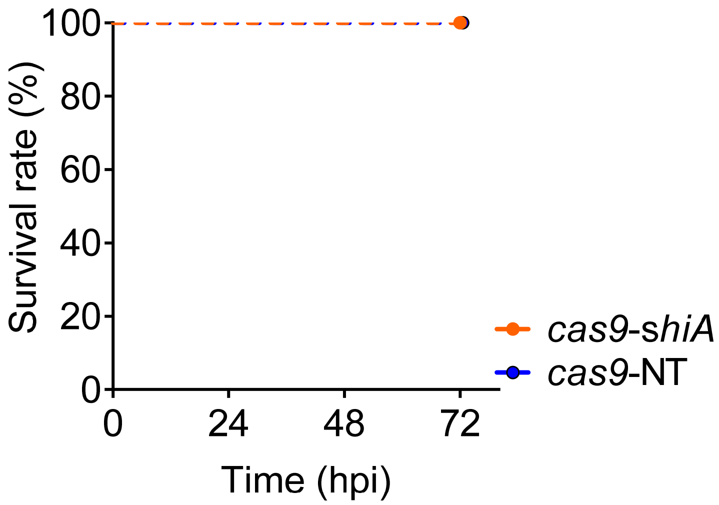

Supplementary Figure 4: **Injection of *cas9* phagemids does not affect zebrafish viability.** Survival curve of zebrafish larvae injected with ~10^4 P1 transducing units of *cas9-shiA* phagemid (orange) or *cas9*-NT phagemid (blue) without administering a lethal dosage of *S. flexneri*, incubated at 32.5°C for 72 hours post-infection (hpi). Without *S. flexneri* infection, zebrafish larvae showed a 100 % survival rate at all hpi after *shiA*-targeting and non-targeting phagemid treatments, suggesting that both the Cas9-killing effect and lysates cytotoxicity were specific towards *S. flexneri*. Non-significant difference in survival was tested using a Log-rank (Mantel-Cox) test ($p > 0.05$).

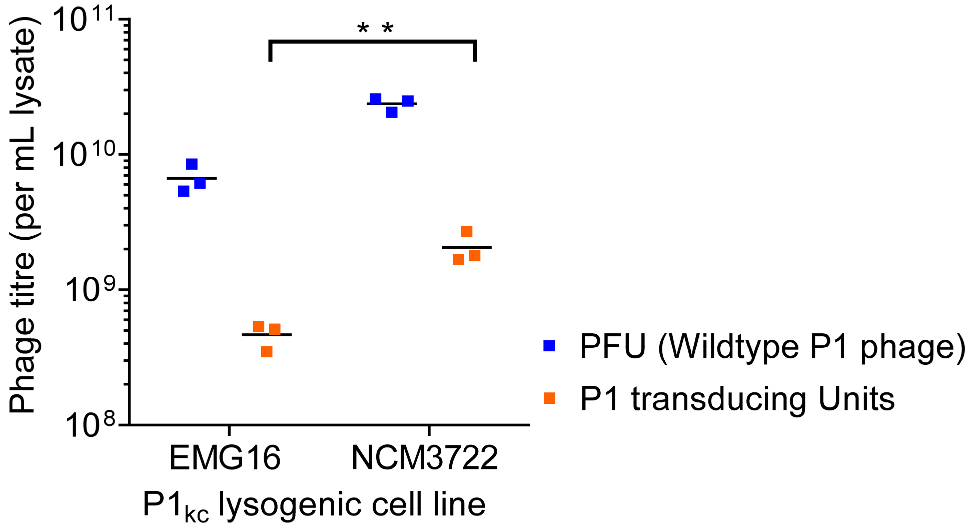

Supplementary Figure 5: NCM3722 P1 lysogen gave a higher phage titre as compared to lysates prepared from EMG16 P1 lysogen. Crude lysates were prepared from NCM3722 and EMG16 harbouring wildtype $P1_{kc}$. Quantification of plaque forming units (in blue) and chloramphenicol-resistant CFU (in orange), representing wildtype P1 phage and P1 transducing unit titres respectively, were carried out on naïve NCM3722 host cells. Crude phage lysates prepared from wildtype P1 lysogenic *E. coli* cell line gave

approximately 10 to 15 wildtype P1 bacteriophage per P1 phagemid packaged into transducing unit (Westwater et al., 2002). Each data point is represented a single biological repeat with readings from 4 technical repeats. Data is represented in the form of mean ± SEM. The p-value was determined using a Kruskal-Wallis test with significance defined by $p < 0.05$. ** represents $p < 0.005$, for comparison of phagemid transducing units between lysates prepared from EMG16 and NCM3722 P1 lysogen.

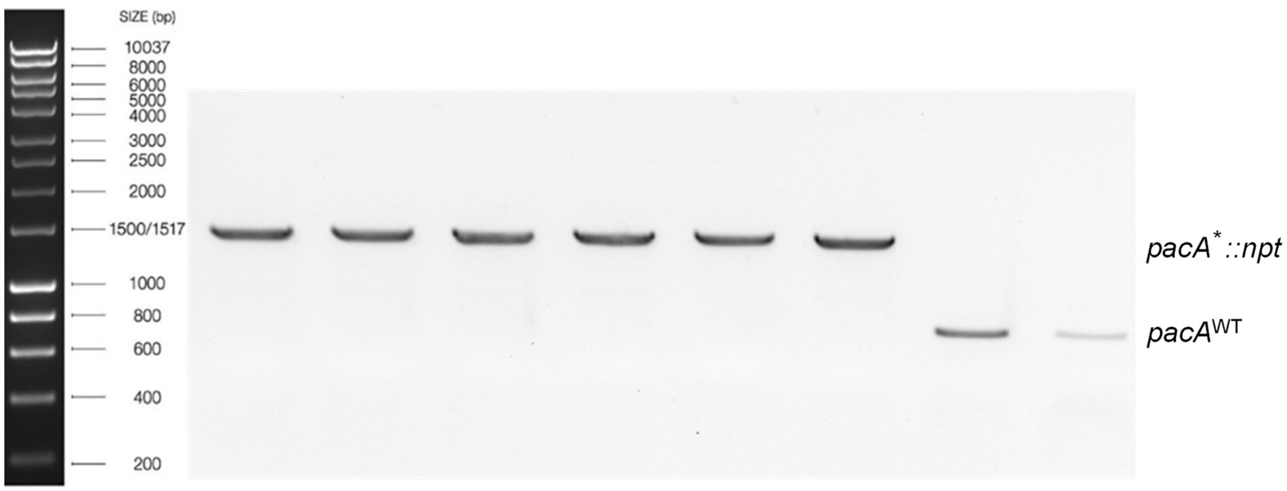

Supplementary Figure 6: Gel Image showing gel electrophoresis results of the colony PCR reactions, carried out on *pacA ::*npt* EMG16 P1 lysogen and wildtype EMG16 P1 lysogen.** *pacA* modified P1 lysogen contains the additional ≈ 850 bps DNA fragment as compared to wildtype P1 lysogen.

Supplementary Table 1: Bacterial strains used in this study

Strain	Description	Remarks/source
EMG16 *E. coli* K-12 C600 $P1_{kc}$	*E. coli* P1 lysogen used for phage lysate preparation	CGSC#: 4405
EMG16 *E. coli* K-12 C600 *pacA*$^-$::*npt*(kan^R) $P1_{kc}$	*E. coli pacA* modified, P1 lysogen with kanamycin resistance cassette retained on the P1 bacteriophage genome	Strain generated in this study
E. coli NCM3722	Used for phage lysate preparation, transduction assay and plaque forming units (PFU) quantification	CGSC#: 12355
E. coli MC1061::*npt*	*E. coli* that carried a chromosomal copy of the *npt* (neomycin phosphotransferase) antibiotic resistance gene	Strain generated in this study
E. coli MC1061	Used as a control to assess the specificity of the chromosomal targeting effect of *cas9-npt* phagemid	CGSC#: 6649
E. coli TOP10	Used for routine molecular cloning, assessment of phagemid transduction efficiency	Thermo Fisher Scientific, Waltham, MA, USA
E. coli BL21	Used for assessment of phagemid transduction efficiency	Thermo Fisher Scientific, Waltham, MA, USA
E. coli K-12 MG1655	Used for assessment of phagemid transduction efficiency	CGSC#: 6300
S. flexneri serotype 2a 2457O	Avirulent mutant which contains a transposon insertion in the major-virulence plasmid disrupts *virF* and subsequently prevents activation of the *Ipa* genes essential for bacterial invasion of the host. Used for preliminary assessment of spacer sequence mediated lethality of P1 J72114 phagemids	ATCC: 29903
S. flexneri serotype *5a* M90T GFP	An Ampicillin resistant, virulent strain of *S. flexneri* expressing GFP, used for *in vitro* and *in vivo* assessment of spacer sequence mediated lethality of P1 J72114 phagemids	Mostowy *et al.,* 2010[4]

Supplementary Table 2 Plasmids and phagemids used in this study

Plasmid/phagemid	Description	Remarks/source
BBa_J72114-BBa_J72100	complete, arabinose inducible phagemid, chloramphenicol resistant, p15a vector, with constitutive *lacZ* expression	Original phagemid construct, a gift from Christopher Anderson (Addgene plasmid #40781)).
BBa_J72114.J23115.*gfp*	Complete J72114 phagemid with constitutive promoter Bba_J23115 and RBS Bba_B0030 regulating GFP expression.	Phagemid generated in this study
pACYC184-pCas9	Bacterial expression of Cas9 nuclease, tracrRNA and crRNA guide from *S. pyogenes*	A gift from Luciano Marraffini (Addgene plasmid # 42876)
BBa_J72114.*cas9*	Complete J72114 phagemid with constitutive *cas9* expression, tracrRNA and crRNA guide from *S. pyogenes* derived from p*cas9*	Phagemid generated in this study
Bba_J72114-pBBR1-c*as9*	Complete J72114.*cas9* phagemid with pBBR1 origin of replication	Used for both *in vitro* and *in vivo* transduction of *S. flexneri* cells
pkD46	Lambda red-mediated recombineering, used for genetic modification of *pacA* gene of P1 bacteriophage	CGSC
pSC101.*pacA**::*npt*	*pacA* genetic modification template with kanamycin resistance cassette, pSC101 origin of replication	Plasmid generated in this study

Supplementary Table 3: DNA sequences of constructs used in this study

DNA sequence of J72114 phagemid[2]

Lp_{Sit} regulating *pacA* expression[2]

gaatctggtgtgttaaccgattctacgagtagtcatttgttcattgagtaggaatattgttg

pacA gene sequence with its native RBS[2]

cgaaaggaagcataagtgacctgggacgatcacaagaagaatttgctcgcctggcgcgagatggtggttacaccatcgcacagtatgccgccg
agtttaatcttaaccctaataccgcacgtcgttatctccgtgccttcaaagaagacaccaggactacggacagccgcaagccaaataagccagtca
ggaagccactaaaaagcatgatcattgatcactctaatgatcaacatgcaggtgatcacattgcggctgaaatagcggaaaaacaaagagttaat
gccgttgtcagtgccgcagtcgagaatgcgaagcgccaaaataagcgcataaatgatcgttcagatgatcatgacgtgatcacccgcgcccaccg
gaccttacgtgatcgcctggaacgcgacaccctggatgatgatggtgaacgctttgagttcgaagttggcgattacctgatagataacgttgaagcgc
ggaaggccgcgcgcgctatgttgcgtcggtccggggccgatgttctggaaaccactcttctggaaaagtctctttctcatctccttatgctggagaacg
ccagggatacgtgtattcgcctggtgcaggaaatgcgcgatcagcaaaaagacgatgatgaaggtactccgcctgaataccgtatcgcgagcatg
ctaaacagctgttccgcgcagataagcagcctgatcaacaccatttacagcatccggaataactatcgaaaagaaagccgggaggcggaaaag
cacgctttatctatggggcaagctggcattgttaagctggcatacgaacgaaagcgtgaaaataactggtcagtgctggaagcggctgagttcatcg
aggcgcatggaggaaaagtgccgcccctgatgctggagcaaatcaaagccgatctgcgtgctcctaagaccaataccgatgatgaggaaaacc
aaacagcatctggcgctccatcacttgaggatctggataaaatcgcgcgagaacgggccgccagccgccgcgctgatgccgcattgtggattgag
catcgtagagaagaaattgccgatatcgtcgatacaggtggttatggtgatgtcgatgcggaaggcatatcaaacgaagcatggcttgaacaggat
ctggacgaagacgaggaggaagacgaagaagttacccgcaaactgtacggggatgatgattaa

Non-coding *repL* sequence (without start codon)[2]

ttaccctctgaatcctgccggtataccccattgttcgttatctttatttttggctaaaaccgcattaagagcttcgtttaccgtcatgcaatgcggtaggttatc
gaagtttgatatcccgccaatatcaggcgaacgcttgttTttcaggtaagcatatttccgcgcagccgcctctactttctgcttgaactcatgtttttgagtg
cgtttttggataaccgcagattgtcagcctttgcttttgccttagcgatccatgaagtcaatttttgaggctggttgttccggcaccgccggaaactgatct
ttttgttttttaacttgtgacttcttattctttattgccacgtcatcctgacaggggggagggggtatcatttgacatggggggtgtggataaaaaattaaataa
agccaatgtcttagcgagaacagctttaaccttggttgccgctgaCgaaatctttaatttgctttctatcagcgcatttttggcttgttgtgcgaaggccaa
aaaggatggtgtaaaccggtacaggttagcgcgacgttcacggtgatcgccgataacaatctctacagacagaatacctttgtttacagcttcacgg
aatgcacgaacgacggttgattggctataaccagtttctgccgcgatcaggcggtgaggcttgtgaatgaagtattcactggttgttgccgcgagattt
gcacattgcgacaggatatgcccggcgctacgggatagaccggagtgtgttacaaagcaggccaattcatagccagaaaaagtaaaatcgcttta

Non-coding *cin* sequence (without start codon)[2]

ccgagttctcttaaaccaaggtttaggattgaaatgatgacgccggaaacttcttataaagcgtggaaacagccacatcatagatgattgcaacctgc
ttacgggggatgcccttctccagcaatcgccgcatttgctgccatgtttcttcttggtatttaggccgacgcccacctatacgaccttctgcgcgagctgc
atcaagtccagcgcgtgtacgttcaacgataagctcacgttccatttctgccagcgccccccattacgtgaaagaaaaagcgccccattggtgtactg
gtgtcgatggagtcagtgagactccggaagttaatgcctctgtcacgcagctcttccaccagcacaactaagtgacgcatgctgcgcccaagacgg
tctaacttccatacgaccagggtatcacctctggaaagTtacggagtaccttttttaacccagggcgctcagcctttttgccgctcgccttgtcctcaaa
aattagctcacatcctgcgctttcaagagcgtttcgttgtaaagcagtgttttgttcatttgttgatacgcgtacatagcctattag

P_{BAD} promoter regulating *coi* expression[2]

aagaaaccaattgtccatattgcatcagacattgccgtcactgcgtcttttactggctcttctcgctaaccaaaccggtaaccccgcttattaaaagcatt
ctgtaacaaagcgggaccaaagccatgacaaaaacgcgtaacaaaagtgtctataatcacggcagaaaagtccacattgattatttgcacggcgt
cacactttgctatgccatagcatttttatccataagattagcggatcttacctgacgctttttatcgcaactctctactgtttctccat

araC with its native promoter and RBS[2]

ctctgaatggcgggagtatgaaaagtatggctgaagcgcaaaatgatcccctgctgccgggatactcgtttaatgcccatctggtggcgggtttaacg
ccgattgaggccaacggttatctcgatttttttatcgaccgaccgctgggaatgaaaggttatattctcaatctcaccattcgcggtcagggggtggtga
aaaatcagggacgagaatttgtttgccgaccgggtgatattttgctgttcccgccaggagagattcatcactacggtcgtcatccggaggctcgcgaa
tggtatcaccagtgggtttactttcgtccgcgcgcctactggcatgaatggcttaactggccgtcaatatttgccaatacggggttctttcgcccggatga
agcgcaccagccgcatttcagcgacctgtttgggcaaatcattaacgccgggcaaggggaagggcgctattcggagctgctggcgataaatctgc
ttgagcaattgttactgcggcgcatggaagcgattaacgagtcgctccatccaccgatggataatcgggtacgcgaggcttgtcagtacatcagcga
tcacctggcagacagcaattttgatatcgccagcgtcgcacagcatgtttgcttgtcgccgtcgcgtctgtcacatcttttccgccagcagttagggatta
gcgtcttaagctggcgcgaggaccaacgtatcagccaggcgaagctgcttttgagcaccacccggatgcctatcgccaccgtcggtcgcaatgttg
gttttgacgatcaactctatttctcgcgggtatttaaaaaatgcaccggggccagcccgagcgagttccgtgccggttgtgaagaaaaagtgaatgat
gtagccgtcaagttgtcataa

coi with its native RBS[2]

tacagtgaggcataattatggctttcattccaccaaccatcgacgacgttagacattgctctaacgctttatctgtagaccccgccgaaaccgacgctg
cccgcgccattgctgaacactactcaaagatatccaatcaggagtaccgcatcacccaagacgacctggatgatctcactgacacaatcgaatatc
tcatggccactaaccagccagactcacaataa

rrnB T1 terminator after *coi* coding sequence[2]

caaataaaacgaaaggctcagtcgaaagactgggcctttcgttttatctgttgtttgtcggtgaacgctctc

J72114 vector backbone, with p15A *ori*, *cat* gene in blue conferring chloramphenicol resistance[2]

agtcagagtagaatagaagtatcattctcaccaataaaaaacgcccggcggcaaccgagcgttctgaacaaatccagatggagttctgaggtcatt
actggatctatcaacaggagtccaagcgagctcgatatcaaattacgccccgccctgccactcatcgcagtactgttgtaattcattaagcattctgcc
gacatggaagccatcacaaacggcatgatgaacctgaatcgccagcggcatcagcaccttgtcgccttgcgtataatatttgcccatggtgaaaac
gggggcgaagaagttgtccatattggccacgtttaaatcaaaactggtgaaactcacccagggattggctgagacgaaaaacatattctcaataaa
ccctttagggaaataggccaggttttcaccgtaacacgccacatcttgcgaatatatgtgtagaaactgccggaaatcgtcgtggtattcactccaga
gcgatgaaaacgtttcagtttgctcatggaaaacggtgtaacaagggtgaacactatcccatatcaccagctcaccgtctttcattgccatacgaaatt
ccggatgagcattcatcaggcgggcaagaatgtgaataaaggccggataaaacttgtgcttatttttctttacggtctttaaaaaggccgtaatatcca
gctgaacggtctggttataggtacattgagcaactgactgaaatgcctcaaaatgttctttacgatgccattgggatatatcaacggtggtatatccagt
gatttttttctccatttagcttccttagctcctgaaaatctcgataactcaaaaaatacgcccggtagtgatcttatttcattatggtgaaagttggaacctctt
acgtgcccgatcaaagatccgcaccgccggacatcagcgctagcggagtgtatactggcttactatgttggcactgatgagggtgtcagtgaagtg
cttcatgtggcaggagaaaaaaggctgcaccggtgcgtcagcagaatatgtgatacaggatatattccgcttcctcgctcactgactcgctacgctcg
gtcgttcgactgcggcgagcggaaatggcttacgaacggggcggagatttcctggaagatgccaggaagatacttaacagggaagtgagaggg
ccgcggcaaagccgtttttccataggctccgcccccctgacaagcatcacgaaatctgacgctcaaatcagtggtggcgaaacccgacaggacta
taaagataccaggcgtttccccctggcggctccctcgtgcgctctcctgttcctgcctttcggtttaccggtgtcattccgctgttatggccgcgtttgtctca
ttccacgcctgacactcagttccgggtaggcagttcgctccaagctggactgtatgcacgaaccccccgttcagtccgaccgctgcgccttatccggt
aactatcgtcttgagtccaacccggaaagacatgcaaaagcaccactggcagcagccactggtaattgatttagaggagttagtcttgaagtcatgc
gccggttaaggctaaactgaaaggacaagttttggtgactgcgctcctccaagccagttacctcggttcaaagagttggtagctcagagaaccttcg
aaaaaccgccctgcaaggcggttttttcgttttcagagcaagagattacgcgcagaccaaaacgatctcaagaagatcatcttattaatcagataaa
atatttctaaggcctcccctgattctgtggataaccgggatctgtaaggatcaaccactttgtacaagaaagctgggtcgaattgagatccgaacggttt
attacgtacatcaggtaaaactgaccgataagccgctttcttttgggtatagtgtcgtggacagtcattcatctttctgcccctccaaaagtaaaaaccc
gccgaagcgggtttttacgtaaaacaggtgaaactgaccgataagccgctttcttttgggtatagtgtcgtggacagtcattcatctttctgcccctccaa
aagcaaaaacccgccgaagcgggtttttacgtaaaccaggtgaaactgaccgataagccgctttcttttgggtatagcgtcgtggacagtcattcatc
tttccgcccctccaaaagcaaaaacccgccgaagcgggtttttacgtaaatcaggtgaaactgaccgataagccgggttctgtcgtggacagtcatt
catctaggccagcaatcgctcagatcc

DNA sequence of J23110.RBS30.*gfp* construct

J23110

tttacggctagctcagtcctaggtacaatgctagc

RBS30

attaaagaggagaaa

gfp

atgcgtaaaggagaagaacttttcactggagttgtcccaattcttgttgaattagatggtgatgttaatgggcacaaattttctgtcagtggagagggtga
aggtgatgcaacatacggaaaacttacccttaaatttatttgcactactggaaaactacctgttccatggccaacacttgtcactactttcggttatggtgt
tcaatgctttgcgagatacccagatcatatgaaacagcatgactttttcaagagtgccatgcccgaaggttatgtacaggaaagaactatatttttcaa
agatgacgggaactacaagacacgtgctgaagtcaagtttgaaggtgatacccttgttaatagaatcgagttaaaaggtattgattttaaagaagatg
gaaacattcttggacacaaattggaatacaactataactcacacaatgtatacatcatggcagacaaacaaaagaatggaatcaaagttaacttca
aaattagacacaacattgaagatggaagcgttcaactagcagaccattatcaacaaaatactccaattggcgatggccctgtccttttaccagacaa
ccattacctgtccacacaatctgccctttcgaaagatcccaacgaaaagagagaccacatggtccttcttgagtttgtaacagctgctgggattacac
atggcatggatgaactatacaaataa

*pacA** modification template, *lpa* coding sequence in blue, flippase recognition motifs (FRT) in red, kanamycin resistance gene (*npt*) underlined with a continuous line, *pacA* coding sequence underlined with a dotted line, *pac* site is in upper case letters, synonymous *pacA** mutations indicated in red lower cases letters

agatccactctctgaatcctgaattctttcgccgtagccgcgctccgcgccggtttgatgaaaaactccagaacgaactctatatgacgcaggacgaa
aaggaacgccgggagcaccagccttgggtaatggcgcgtcaacttttcaataaggtggcccgtcagcaccgtcattacggtaatgccacatccgca
cgtatctgactgtcaaacatgagaattaattccggggatccgtcgacctgcagttcgaagttcctattctctagaaagtataggaacttcagagcgctttt
gaagctcacgctgccgcaagcactcagggcgcaagggctgctaaaggaagcggaacacgtagaaagccagtccgcagaaacggtgctgacc
ccggatgaatgtcagctactgggctatctggacaagggaaaacgcaagcgcaaagagaaagcaggtagcttgcagtgggcttacatggcgatag
ctagactgggcggttttatggacagcaagcgaaccggaattgccagctggggcgccctctggtaaggttgggaagccctgcaaagtaaactggatg
gctttcttgccgccaaggatctgatggcgcaggggatcaagatctgatcaagagacaggatgaggatcgtttcgcatgattgaacaagatggattgc
acgcaggttctccggccgcttgggtggagaggctattcggctatgactgggcacaacagacaatcggctgctctgatgccgccgtgttccggctgtca
gcgcaggggcgcccggttctttttgtcaagaccgacctgtccggtgccctgaatgaactgcaggacgaggcagcgcggctatcgtggctggccacg
acgggcgttccttgcgcagctgtgctcgacgttgtcactgaagcgggaagggactggctgctattgggcgaagtgccggggcaggatctcctgtcatc
tcaccttgctcctgccgagaaagtatccatcatggctgatgcaatgcggcggctgcatacgcttgatccggctacctgcccattcgaccaccaagcga
aacatcgcatcgagcgagcacgtactcggatggaagccggtcttgtcgatcaggatgatctggacgaagagcatcaggggctcgcgccagccga
actgttcgccaggctcaaggcgcgcatgcccgacggcgaggatctcgtcgtgacccatggcgatgcctgcttgccgaatatcatggtggaaaatgg
ccgcttttctggattcatcgactgtggccggctgggtgtggcggaccgctatcaggacatagcgttggctacccgtgatattgctgaagagcttggcggc
gaatgggctgaccgcttcctcgtgctttacggtatcgccgctcccgattcgcagcgcatcgccttctatcgccttcttgacgagttcttctaataactcggta
ccaaattccagaaaagaggcctcccgaaaggggggccttttttcgttttggtccggggatcttgaagttcctattccgaagttcctattctctagaaagtat
aggaacttcgaagcagctccagcctacacttgattgcttgcccgttccgggccttttgacatgtgactttcgttaccctcgcgtcaaaaagagtttttacga
aaggaagcataagtgacctgggacgatcacaagaagaattttgctcgcctggcgcgagatggtggttacaccatcgcacagtatgccgccgagttt
aatcttaaccctaataccgcacgtcgttatctccgtgccttcaaagaagacaccaggactacggacagccgcaagccaaataagccagtcaggaa
gccactaaaaagcATGATTATCGACCATTCTAAcGAcCAACATGCAGGcGAcCACATTGCGGCTGAAATtGC
GGAAAAgCAgcGtGTcAATGCCGTTGTCAGTGCCGCAGTCGAGAATGCGAAGCGCCAAAATAAGCGCA
TtAAcGAcCGTTCAGAcGAcCATGACGTAATTACCcgcgcccaccggaccttacgtgatcgcctggaacgcgacaccctggat
gatgatggtgaacgctttgaattcgaagttggcgattacctgatagataacgttgaagcgcggaaggccgcgcgcgctatgttgcgtcggtccggggc
cgatgttctggaaaccactcttctggaaaagtctctttctcatctccttatgctggagaac

DNA sequence of CRISPR Cas9 construct from pCas9 plasmid (Addgene plasmid #42876)
Promoter sequence of *trans*-activating *crRNA*<br><br>agtattaagtattgttttatggctgataaatttctttgaatttctccttgattatttgttataaaagttataaaataatcttgtt
Trans-activating crRNA<br><br>ggaaccattcaaaacagcatagcaagttaaaataaggctagtccgttatcaacttgaaaaagtggcaccgagtcggtgc
Promoter and RBS regulating *cas9* expression<br><br>atagaatgataacaaaataaactactttttaaaagaattttgtgttataatctatttattattaagtattgggtaatattttttgaagagatattttgaaaaaga<br>aaaattaaagcatattaaactaatttcggaggtcattaaaactattattgaaatcatcaaactcattatggatttaatttaaactttttattttaggaggcaaa<br>a
crRNA guide leader sequence in upper case letters, direct repeats in blue and protospacer sequence in black, BsaI sites are underlined.<br><br>TATTTCTTAATAACTAAAAATATGGTATAATACTCTTAATAAATGCAGTAATACAGGGGCTTTTCAAGA<br>CTGAAGTCTAGCTGAGACAAATAGTGCGATTACGAAATTTTTTAGACAAAAATAGTCTACGAGgttttaga<br>gctatgctgttttgaatggtcccaaaactgagaccagtctcggaagctcaaaggtctcgttttagagctatgctgttttgaatggtcccaaaac

cas9, DNA in upper case represent the sequence between *cas9* ORF and the crRNA leader sequence

atggataagaaatactcaataggcttagatatcggcacaaatagcgtcggatgggcggtgatcactgatgaatataaggttccgtctaaaaagttcaa
ggttctgggaaatacagaccgccacagtatcaaaaaaaatcttataggggctcttttatttgacagtggagagacagcggaagcgactcgtctcaaa
cggacagctcgtagaaggtatacacgtcggaagaatcgtatttgttatctacaggagatttttcaaatgagatggcgaaagtagatgatagtttctttcat
cgacttgaagagtctttttggtggaagaagacaagaagcatgaacgtcatcctatttttggaaatatagtagatgaagttgcttatcatgagaaatatcc
aactatctatcatctgcgaaaaaaattggtagattctactgataaagcggatttgcgcttaatctatttggccttagcgcatatgattaagtttcgtggtcattt
tttgattgagggagatttaaatcctgataatagtgatgtggacaaactatttatccagttggtacaaacctacaatcaattatttgaagaaaaccctattaa
cgcaagtggagtagatgctaaagcgattctttctgcacgattgagtaaatcaagacgattagaaaatctcattgctcagctccccggtgagaagaaaa
atggcttatttgggaatctcattgctttgtcattgggtttgacccctaattttaaatcaaattttgatttggcagaagatgctaaattacagctttcaaaagatac
ttacgatgatgatttagataatttattggcgcaaattggagatcaatatgctgatttgtttttggcagctaagaatttatcagatgctattttactttcagatatcc
taagagtaaatactgaaataactaaggctcccctatcagcttcaatgattaaacgctacgatgaacatcatcaagacttgactcttttaaaagctttagtt
cgacaacaacttccagaaaagtataaagaaatctttttgatcaatcaaaaaacggatatgcaggttatattgatgggggagctagccaagaagaatt
ttataaatttatcaaaccaattttagaaaaaatggatggtactgaggaattattggtgaaactaaatcgtgaagatttgctgcgcaagcaacggaccttt
gacaacggctctattccccatcaaattcacttgggtgagctgcatgctattttgagaagacaagaagacttttatccatttttaaaagacaatcgtgagaa
gattgaaaaaatcttgacttttcgaattccttattatgttggtccattggcgcgtggcaatagtcgttttgcatggatgactcggaagtctgaagaaacaatt
accccatggaattttgaagaagttgtcgataaaggtgcttcagctcaatcatttattgaacgcatgacaaactttgataaaaatcttccaaatgaaaaag
tactaccaaaacatagtttgctttatgagtattttacggtttataacgaattgacaaaggtcaaatatgttactgaaggaatgcgaaaaccagcatttctttc
aggtgaacagaagaaagccattgttgatttactcttcaaaacaaatcgaaaagtaaccgttaagcaattaaaagaagattatttcaaaaaaatagaa
tgttttgatagtgttgaaatttcaggagttgaagatagatttaatgcttcattaggtacctaccatgatttgctaaaaattattaaagataaagatttttggata
atgaagaaaatgaagatatcttagaggatattgttttaacattgaccttatttgaagatagggagatgattgaggaaagacttaaaacatatgctcacct
ctttgatgataaggtgatgaaacagcttaaacgtcgccgttatactggttggggacgtttgtctcgaaaattgattaatggtattagggataagcaatctg
gcaaaacaatattagatttttgaaatcagatggttttgccaatcgcaattttatgcagctgatccatgatgatagtttgacatttaaagaagacattcaaaa
agcacaagtgtctggacaaggcgatagtttacatgaacatattgcaaatttagctggtagccctgctattaaaaaaggtattttacagactgtaaaagtt
gttgatgaattggtcaaagtaatggggcggcataagccagaaaatatcgttattgaaatggcacgtgaaaatcagacaactcaaaagggccagaa
aaattcgcgagagcgtatgaaacgaatcgaagaaggtatcaaagaattaggaagtcagattcttaaagagcatcctgttgaaaatactcaattgcaa
aatgaaaagctctatctctattatctccaaaatggaagagacatgtatgtggaccaagaattagatattaatcgtttaagtgattatgatgtcgatcacatt
gttccacaaagtttccttaaagacgattcaatagacaataaggtcttaacgcgttctgataaaaatcgtggtaaatcggataacgttccaagtgaagaa
gtagtcaaaaagatgaaaaactattggagacaacttctaaacgccaagttaatcactcaacgtaagtttgataatttaacgaaagctgaacgtggag
gtttgagtgaacttgataaagctggttttatcaaacgccaattggttgaaactcgccaaatcactaagcatgtggcacaaattttggatagtcgcatgaat
actaaatacgatgaaaatgataaacttattcgagaggttaaagtgattaccttaaaatctaaattagtttctgacttccgaaaagatttccaattctataaa
gtacgtgagattaacaattaccatcatgcccatgatgcgtatctaaatgccgtcgttggaactgctttgattaagaaatatccaaaacttgaatcggagttt
gtctatggtgattataaagtttatgatgttcgtaaaatgattgctaagtctgagcaagaaataggcaaagcaaccgcaaaatatttcttttactctaatatca
tgaacttcttcaaaacagaaattacacttgcaaatggagagattcgcaaacgccctctaatcgaaactaatggggaaactggagaaattgtctggga
taaagggcgagattttgccacagtgcgcaaagtattgtccatgccccaagtcaatattgtcaagaaaacagaagtacagacaggcggattctccaa
ggagtcaattttaccaaaaagaaattcggacaagcttattgctcgtaaaaaagactgggatccaaaaaaatatggtggttttgatagtccaacggtag
cttattcagtcctagtggttgctaaggtggaaaaagggaaatcgaagaagttaaaatccgttaaagagttactagggatcacaattatggaaagaagt
tcctttgaaaaaaatccgattgactttttagaagctaaaggatataaggaagttaaaaaagacttaatcattaaactacctaaatatagtctttttgagtta
gaaaacggtcgtaaacggatgctggctagtgccggagaattacaaaaaggaaatgagctggctctgccaagcaaatatgtgaatttttatatttagct
agtcattatgaaaagttgaagggtagtccagaagataacgaacaaaaacaattgtttgtggagcagcataagcattatttagatgagattattgagca
aatcagtgaattttctaagcgtgttattttagcagatgccaatttagataaagttcttagtgcatataacaaacatagagacaaaccaatacgtgaacaa
gcagaaaatattattcatttatttacgttgacgaatcttggagctcccgctgcttttaaatattttgatacaacaattgatcgtaaacgatatacgtctacaaa
agaagttttagatgccactcttatccatcaatccatcactggtctttatgaaacacgcattgatttgagtcagctaggaggtgactgaAGTATATTTT
AGATGAAGAT

Supplementary Table 4: Primers for cloning the spacer sequences of *cas9* phagemid crRNAs

Name of sequence(s)	Sequence (5' to 3')[a]	Remarks
npt forward	AAAC**TTCATCGACTGTGGCCGGCT**G	Chromosomal targeting of *npt* gene of *E. coli* MC1061::*npt*
npt reverse	AAAAC**AGCCGGCCACAGTCGATGAA**	Chromosomal targeting of *npt* gene of *E. coli* MC1061::*npt*
sigA forward (G30)	AAAC**ACGACTTTCCCAGTCGGGCT**G	Chromosomal targeting of *sigA* gene of *S. flexneri* (crRNA termed G30)
sigA reverse (G30)	AAAAC**AGCCCGACTGGGAAAGTCGT**	Chromosomal targeting of *sigA* gene of *S. flexneri* (crRNA termed G30)
pic forward (G31)	AAAC**GCTTCAGCATTGTTTGAGTC**G	Chromosomal targeting of *pic* gene of *S. flexneri* (crRNA termed G31)
pic reverse (G31)	AAAAC**GACTCAAACAATGCTGAAGC**	Chromosomal targeting of *pic* gene of *S. flexneri* (crRNA termed G31)
shiD forward (G34)	AAAC**AATTTCTACTGATTAGATTA**G	Chromosomal targeting of *shiD* gene of *S. flexneri* (crRNA termed G34)
shiD reverse (G34)	AAAAC**TAATCTAATCAGTAGAAATT**	Chromosomal targeting of *shiD* gene of *S. flexneri* (crRNA termed G34)
shiA forward (G37)	AAAC**GCATGACTTCTCCGGCTCTC**G	Chromosomal targeting of *shiA* gene of *S. flexneri* (crRNA termed G37)
shiA reverse (G37)	AAAAC**CGAGAGCCGGAGAAGTCATG**	Chromosomal targeting of *shiA* gene of *S. flexneri* (crRNA termed G37)

[a]DNA sequences in bold represent the 20 bp spacer sequences.

References

1. Westwater, C., Schofield, D.A., Schmidt, M.G., Norris, J.S. and Dolan, J.W. (2002) Development of a P1 phagemid system for the delivery of DNA into Gram-negative bacteria. *Microbiology*, **148**, 943–950.

2. Kittleson, J.T., Deloache, W., Cheng, H.Y. and Anderson, J.C. (2012) Scalable plasmid transfer using engineered P1-based phagemids. *ACS Synth. Biol.*, **1**, 583–589.

3. Heinzel, T., Velleman, M. and Schuster, H. (1990) The c1 repressor inactivator protein coi of bacteriophage P1. Cloning and expression of coi and its interference with c1 repressor function. *J. Biol. Chem.,* **265**, 17928-17934.

4. Mostowy, S., Bonazzi, M., Hamon, M., Tham, T., Mallet, A., Lelek, M., Gouin, E., Demangel, C., Brosch, R. and Zimmer, C. *et al.* (2010) Entrapment of intracytosolic bacteria by septin cage-like structures. *Cell Host Microbe*, **8**, 433-444.
